# Fabrication and Plasma Modification of Nanofibrous Tissue Engineering Scaffolds

**DOI:** 10.3390/nano10010119

**Published:** 2020-01-08

**Authors:** Mahtab Asadian, Ke Vin Chan, Mohammad Norouzi, Silvia Grande, Pieter Cools, Rino Morent, Nathalie De Geyter

**Affiliations:** 1 Research Unit Plasma Technology (RUPT), Department of Applied Physics, Ghent University, Sint-Pietersnieuwstraat 41, B4, B-9000 Ghent, Belgium; KeVin.chan@UGent.be (K.V.C.); Silvia.Grande@Ugent.be (S.G.); Pieter.Cools@UGent.be (P.C.); Rino.Morent@UGent.be (R.M.); Nathalie.DeGeyter@UGent.be (N.D.G.); 2 Department of Biomedical Engineering, University of Manitoba, Winnipeg, MB R3E 0Z3, Canada; norouzim@myumanitoba.ca

**Keywords:** electrospun nanofibers, non-thermal plasma treatment, tissue engineering

## Abstract

This paper provides a comprehensive overview of nanofibrous structures for tissue engineering purposes and the role of non-thermal plasma technology (NTP) within this field. Special attention is first given to nanofiber fabrication strategies, including thermally-induced phase separation, molecular self-assembly, and electrospinning, highlighting their strengths, weaknesses, and potentials. The review then continues to discuss the biodegradable polyesters typically employed for nanofiber fabrication, while the primary focus lies on their applicability and limitations. From thereon, the reader is introduced to the concept of NTP and its application in plasma-assisted surface modification of nanofibrous scaffolds. The final part of the review discusses the available literature on NTP-modified nanofibers looking at the impact of plasma activation and polymerization treatments on nanofiber wettability, surface chemistry, cell adhesion/proliferation and protein grafting. As such, this review provides a complete introduction into NTP-modified nanofibers, while aiming to address the current unexplored potentials left within the field.

## 1. Introduction

Injury, trauma or disease can lead to degeneration or damage of tissues in the human body, which requires treatments to initiate their repair and regeneration procedure [1]. Conventionally, the condition is treated by transplanting tissue from one point to another point of the same body (an autograft) or from one body site to another body site of a different patient (a transplant or allograft) [2,3]. Although these treatments have been revolutionary and lifesaving, both techniques involve some major issues. The retrieval of autograft tissue is expensive, painful and often correlated with morbidity of the donor site as a result of infections and hematoma. In the same way, allografts also have serious limitations because of their limited supply and the possibility of disease transmission from donor to patient and tissue rejection by the patient’s immune system [4,5]. The abovementioned problems have led to the emergence of tissue engineering (TE), which aims to repair damaged tissues through tissue regeneration rather than replacing them [6,7].

**Figure 1:**
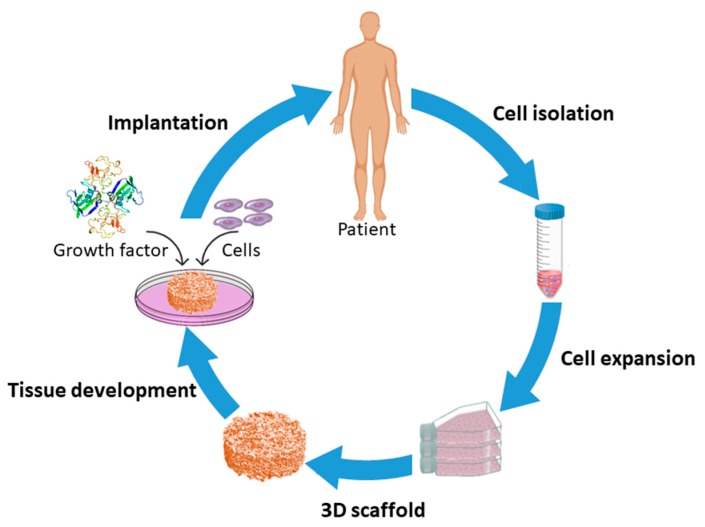
Schematic of the scaffold-based tissue engineering approach.

TE is an interdisciplinary discipline combining the principles of engineering and life sciences to develop biological substitutes that restore, maintain or improve the functions of tissues [1,8]. TE encompasses three major approaches: (1) transplantation of cells isolated from a healthy part to an injured tissue, (2) injection of factors that initiate/induce tissue regeneration like growth factors, differentiation factors, polysaccharides, and peptides to a targeted site; and (3) seeding of cells in combination with growth factors on a three-dimensional (3D) matrix, referred to as “a scaffold”, which acts as a temporary framework on which cells can adhere, grow and differentiate in vitro prior to implantation in vivo [1,9,10,11] as schematically presented in Figure 1. Among these current strategies, scaffold-based TE has become the most commonly used approach and will consequently be the main focus of this review paper.

The design of a scaffold prior to exposure to cells is of vital importance. Although the favorable elements of an engineered scaffold can slightly differ from the tissue characteristics of the target, there are several scaffold requirements which have been identified as crucial:

(1) The scaffold needs to be bio-compatible so that it integrates well with the host body without eliciting any mutagenic, carcinogenic or cytotoxic behavior which can cause a major inflammatory response [12,13,14].
(2) The scaffold must possess the mechanical properties necessary to temporarily offer structural support until new tissue has formed [15,16,17].
(3) The scaffold must possess surface properties that allow attachment, migration, proliferation, and differentiation of cells [18].
(4) The scaffold must be biodegradable in a way that additional surgery is not required for implant removal. Ideally, the degradation rate should match the rate of new tissue formation [19,20].
(5) The porosity of the engineered scaffold and the scaffold’s surface-volume ratio should be high to enable cell attachment, to provide in-growth sites for cells to adhere and proliferate and to facilitate nutrients exchange upon in vitro or in vivo culture [21].
(6) The scaffold should simulate the native extracellular matrix (ECM) both in structure as well as in biological function. The ECM is known to have a fibrillar structure: collagen, the most abundant ECM protein in the human body, is made of continuous fibers with diameters that vary in the ranges of 50 to 500 nm [22,23].

**Figure 2:**
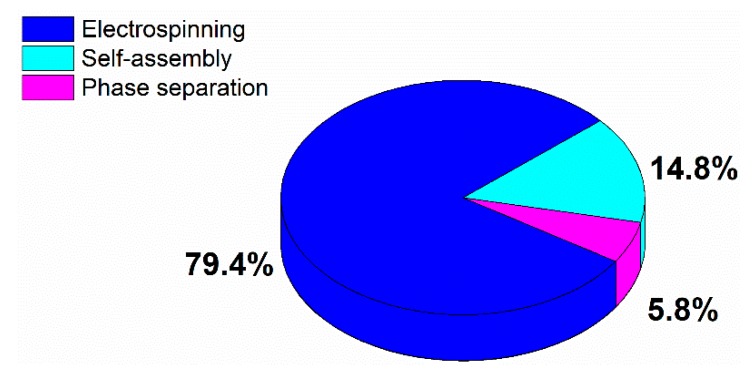
Distribution of publications published worldwide in the period 1994–2019 according to the most common used nanofibrous tissue engineering (TE) scaffold fabrication technique.

TE scaffolds can be fabricated using multiple methods including freeze-drying [24], solvent casting [25], particulate leaching [26], gas foaming [27], rapid prototyping [28,29]. These TE scaffold fabrication methods have already been widely described in multiple review papers and will therefore not be discussed in this paper [24,30,31,32]. All of the above-mentioned methods have their intrinsic advantages and disadvantages, but most of them are incapable of producing ECM mimicking nanofibrous scaffolds. To cope with this issue, researchers have put much effort into developing nanofibrous TE scaffolds, which can mimic the fibrous structure of natural ECM. Currently, there are three different techniques available for the generation of nanofibrous scaffolds: phase separation [33,34], molecular self-assembly [35,36], and electrospinning [37,38]. An introduction to each of these fabrication techniques will be given in the first part of this review paper. Of the mentioned techniques, electrospinning is the most widely studied nanofibrous scaffold fabrication method as evidenced by a survey of publications published worldwide since 1994 (see Figure 2, comparing the frequently used methods to produce nanofibers for tissue engineering applications based on a search in Web of Science), and also seems to hold the most promising results for TE applications. On the contrary, nanofibers synthesized by self-assembly and phase separation have had relatively limited studies that explored their application as scaffolds for TE. As such, this review paper will particularly focus on electrospinning as a nanofibrous TE scaffold fabrication method. 

While electrospinning is capable of constructing nanofibrous TE scaffolds, the ultimate success of these scaffolds is of course also strongly determined by the material selection. Indeed, the material choice influences important scaffold characteristics such as mechanical strength, biocompatibility, and degradation kinetics. Although electrospun fibers can be generated from different material classes including polymers [39], ceramics [40] and inorganics [41], polymers are the primary class of materials for designing electrospun scaffolds thanks to their design flexibility and their excellent bulk properties. As a result, electrospun TE scaffolds have been mostly fabricated from a multitude of both synthetic and natural polymers [42,43]. Natural polymers, owing to their biodegradability and excellent bioactive properties, have attracted significant attention in TE applications. Natural polymers are either compounds of the native ECM or polymers extracted from other biological systems [44,45]. Unfortunately, the applicability of these natural polymers as such is often hindered due to low availability, batch-to-batch variation, weak mechanical properties and rapid degradation in aqueous conditions [46]. In the last few decades, synthetic polymers have been developed as alternatives to natural polymers due to their improved performance, low cost, ease of production and more reproducible properties [47,48]. In the field of electrospun TE scaffolds, mainly synthetic biodegradable aliphatic polyesters are considered to be excellent scaffold candidates, as their physical and chemical properties, such as mechanical strength and degradation rate, can be tailored to meet specific requirements of TE scaffolds [49,50]. An overview of biodegradable polyesters frequently used for the fabrication of electrospun TE scaffolds together with their most important properties will, therefore, be given in the second part of this review paper. This overview will allow the reader to obtain a basic understanding of the different biodegradable polyesters and will enable him/her to select the most appropriate synthetic polymer for a particular TE application. 

## 2. Fabrication of Nanofibrous TE Scaffolds

As mentioned in the introduction, three methods are currently available for the fabrication of nanofibrous TE scaffolds: (1) phase separation, (2) molecular self-assembly, and (3) electrospinning. Each of these methods will be described in detail in the following paragraphs. As electrospinning is by far the most established method (see Figure 2), most attention will be paid to this nanofibrous TE scaffold fabrication technique.

### 2.1. Thermally-Induced Phase Separation

An interesting method used to manufacture nanofibrous TE scaffolds is phase separation. A phase separation process can be initiated either thermally or by a non-solvent and has already been widely used for the fabrication of porous membranes or foams for filtration and separation methods [51,52]. Non-solvent induced phase separation typically results in matrices with a heterogeneous pore structure, which is not desirable for TE scaffolds as these generally require a uniform pore structure [53,54]. As such, only thermally-induced phase separation will be described in this paper as this technique is capable of producing homogeneous porous scaffolds [55,56].

**Figure 3:**
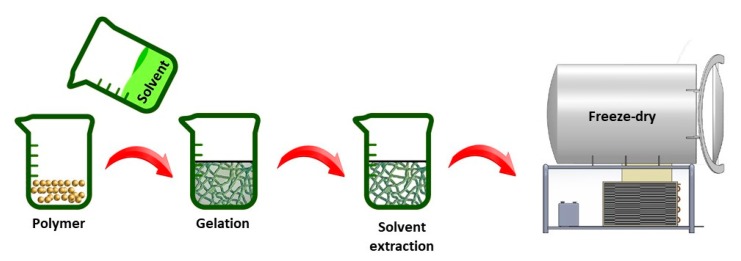
Illustration of thermally-induced phase separation (TIPS) production steps.

When a homogenous polymer solution becomes thermally unstable when applying certain temperature conditions, a multiphase system containing a polymer-rich and a polymer-poor phase can be generated. This is referred to as a thermally-induced phase separation (TIPS) process [57,58]. After solvent removal, the polymer-rich phase will form a 3D matrix with the polymer-poor phase constituting the pores of the matrix. The process typically consists of five different steps: (1) dissolution of the raw polymer in an appropriate solvent, (2) phase separation and gelation, (3) solvent extraction from the gel (polymer-rich phase), (4) freezing and (5) drying (see Figure 3) [34,59]. The porosity and fiber size of the fabricated TE scaffolds can be tuned by controlling different process parameters including polymer/solvent system, polymer concentration, gelation temperature and gelation duration [60,61].

**Figure 4:**
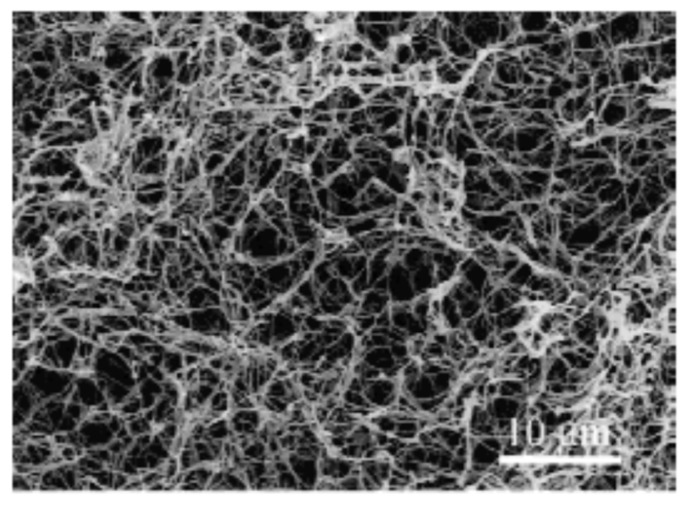
Scanning electron microscopy (SEM) micrograph of poly-l-lactic acid (PLLA) nanofibrous meshes prepared from a 5 *w/v*% PLLA/tetrahydrofuran (THF) solution at 8 °C—reproduced. Reproduced with permission from [62], Copyright Wiley, 1999.

Using TIPS, 3D nanofibrous scaffolds have been first prepared from poly-l-lactic acid (PLLA) in 1999 by Ma et al. [62]. In this pioneering study, PLLA was dissolved in tetrahydrofuran (THF) and the effect of polymer concentration and gelation temperature was studied in detail. The applied gelation duration depended on polymer concentration and gelation temperature and was therefore varied between 5 min and 24 h. A scanning electron microscopy (SEM) micrograph of the scaffold gained in 5 *w/v*% polymer concentration, a gelation temperature of 8 °C and a gelation duration of 25 min can be seen in Figure 4. This image clearly shows the fabrication of a 3D-interconnected randomly oriented fibrous PLLA network with fiber diameters of 160–170 nm.

Results obtained by Ma et al. also revealed that such a 3D nanofibrous network was only obtained from a 5 *w/v*% PLLA/THF solution at low temperatures of gelation (15 °C, 8 °C, −18 °C and −195 °C). The fiber diameter was not strongly affected by the gelation temperature as it was found to be in the range 160–170 nm for all gelation temperatures under study. However, the inter-fiber spacing was observed to be more uniform at lower gelation temperatures. At a gelation temperature of −18 °C, the effect of PLLA concentration (1.0 to 7.5 *w/v*%) was also examined in detail. It was observed that the fiber diameter did not statistically change with polymer concentration (160–170 nm). At low PLLA concentrations, relatively large voids were however acquired characterized by non-uniform inter-fiber spacing, while through enhancing the PLLA concentration, the pore structure became growingly homogenous and symmetrical, with the presence of smaller pores. Indeed, the porosity was found to decrease from 98.5% to 93.8% with increasing PLLA concentration. Later on, in 2004, Yang et al. [63] reported results similar to these of Ma et al. as these authors also showed the formation of porous nanofibrous scaffolds generated by TIPS from low concentration PLLA/THF solutions (2 to 7 *w/v*%). In this case, phase separation was performed at −30 °C for 2 h. Additionally, the authors also observed that when the PLLA concentration was further increased to 9 *w/v*%, scaffolds similar to a piece of rigid sheet without any pores were obtained. The authors also confirmed other conclusions of Ma et al. as they also observed a stable fiber diameter and a decreasing porosity with increasing PLLA concentration.

Unfortunately, making use of the system PLLA/THF, the nanofibrous scaffolds were found to suffer from small size (<10 µm) micropores as in this case it was very hard to simultaneously fabricate nanofibers and a macro/microporous structure. These small size micropores strongly hamper the application of these scaffolds in TE as the scaffold pores need to be sufficiently large to allow cell ingrowth and nutrient exchange. To cope with this issue, He et al. [64] decided to examine a novel ternary system consisting of PLLA/dioxane/water. The authors observed that compressed and uniform nanofibers were fabricated using a PLLA/THF system after gelation at −30 °C for 2 h. In this case, micropores were clearly visible but unfortunately, their diameter was found to be below 10 µm. In contrast, a scaffold that is characterized by random, interconnected micropores and a nanofibrous mesh could be fabricated from a PLLA/dioxane/water (88/12 *v/v*) solution at a 12 °C gelation temperature for 2 h. The average pore size in this case was found to be 50 µm, which is much larger than the pore size of the scaffolds made from PLLA/THF. The micropore walls were found to be made up of PLLA nanofibers with fiber diameters varying from 50 nm to 200 nm. It was thus concluded that a porous scaffold with a nanofibrous structure and large pore sizes suitable for TE applications could be obtained by phase separation of a PLLA/dioxane/water system. 

**Figure 5:**
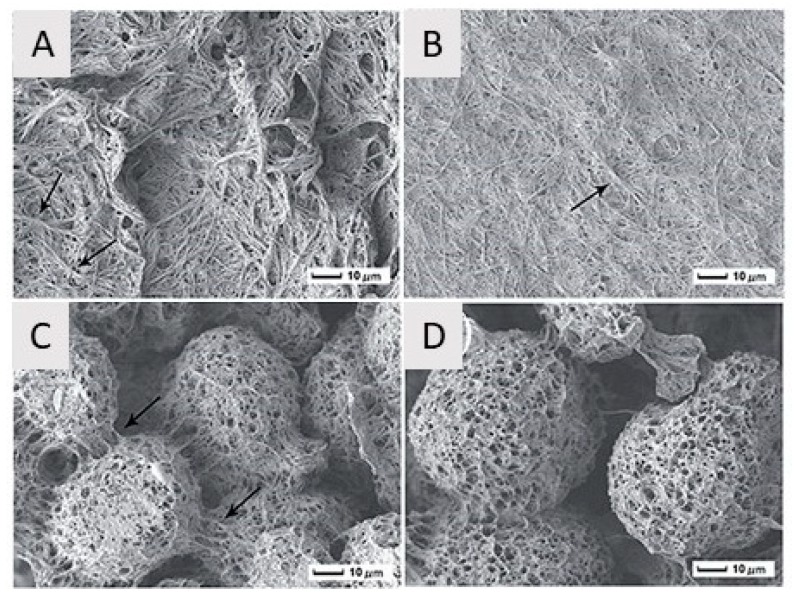
SEM images of PLLA nanofibers and microspheres obtained from PLLA/dimethylformamide (DMF) solutions as a function of PLLA concentration: (**A**) 1 *w/v*%, (**B**) 3 *w/v*%, (**C**) 5 *w/v*% and (**D**) 7 *w/v*% (quenching time: 10 min; crystallization temperature: −10 °C; scale bar: 10 µm)—reproduced with permission from [65], Copyright Royal Sociaty of chemistry, 2015.

Besides the mixture of dioxane/water, also the potential of *N,N*-dimethylformamide (DMF) as a solvent for PLLA has been examined in TIPS experiments. Figure 5 shows SEM images of PLLA nanostructures obtained from PLLA/DMF solutions as a function of PLLA concentration for a gelation process of 10 min at −10 °C. At a low PLLA concentration of 1 *w/v*%, very densely packed, randomly organized long PLLA nanofibers with an average fiber diameter of 110–180 nm were obtained. When the PLLA concentration was increased to 3 *w/v*%, a PLLA fibrous mat composed of disordered nanofibers was still obtained, however, some of the nanofibers tend to bundle together to form a sheaf-like structure (indicated by an arrow in Figure 5B). When the concentration was further increased to 5 and 7 *w/v*%, the scaffold morphology changed to highly porous microspheres with a diameter of 30–70 µm, composed of thin fluffy PLLA nanofibers (average diameter = 150 nm) [65].

Besides PLLA scaffolds, which have been the most widely fabricated using TIPS, nanofibrous scaffolds have also been successfully produced making use of TIPS from gelatin [33,57]. In this case, the addition of ethanol to an aqueous gelatin solution was found to be a requisite to create nanofibrous structures. It was also observed that only when the ethanol/water ratio in the solvent mixture was maintained between 20/80 (*v/v*) and 50/50 (*v/v*), nanofibrous architectures could be created. Using gelatin concentrations between 5 and 10 *w/v*%, the authors were able to generate nanofibrous scaffolds consisting of 160–170 nm thick gelatin nanofibers possessing a porosity between 96 and 98%, which is quite similar to the structural parameters of scaffolds obtained from PLLA. A few other solvent mixtures such as acetone/water, dioxane/water, and THF/water were also examined but were found to be incapable of generating nanofibrous structures [33].

Besides PLLA and gelatin, also poly-ε-caprolactone (PCL) has been fabricated into nanofibrous scaffolds making use of TIPS [66]. PCL was dissolved in a mixture of dioxane and water (mass ratio 90/10) at 40 °C to prepare a 10 *w/v*% PCL solution, after which the solution was cooled to different gelation temperatures ranging from −8 to 12 °C. Results revealed that completely different scaffold morphologies were obtained depending on the gelation temperature. At gelation temperatures ≤4 °C, microspheres formed the matrices and the micropores walls in the microspheres consist of nanofibrous structures similar to what was observed for high concentration PLLA/DMF solutions. In contrast, at high gelation temperatures (≥8 °C), open microporous structures with solid pore walls were obtained. Besides gelation temperature, also the mass ratio of the solvent mixture was found to play a crucial role as with increasing water amount, the nanofibrous assembly was found to disappear [66]. Very recently, highly porous nanofibrous membranes were also successfully fabricated through low temperature-induced phase separation of a chitosan solution in a tertiary solvent mixture (acetic acid/water/ethanol) [67]. Unlike crystalline PLLA, which can organize into nanofibrous structures in a gelation duration of only 10 min, the assembly of semi-crystalline chitosan into nanofibers is a very slow process with typical gelation duration of 8 to 10 h. In this case, the chitosan concentration was fixed at 2 *w/v*%, the optimum quenching temperature and time were found to be equal to −20 °C to fabricate highly porous nanofibers with diameters of 40–60 nm [67].

TIPS can be combined with other processing techniques, such as granular leaching or solid-free structure fabrication, to produce scaffolds with complicated porous structures and precise morphology of the pores [68,69,70]. For instance, sodium chloride granules with a diameter of 200–450 µm were stirred with a warm solution of poly(lactic-co-glycolic acid) (PLGA) in THF, after which the temperature of the solution was decreased to a gelation temperature (−21 or −70 °C) which was set in advance [68]. The fabricated composite gels were subsequently extracted using cold ethanol and to filter the salt particles and remove the remaining solvent, the obtained gels were washed with deionized water. After freeze-drying, a nanofibrous PLGA scaffold containing macropores, left behind by the leached salt, was obtained. 

To conclude, it can be stated that TIPS is a promising technique for the development of nanofibrous scaffolds containing precise pores, pore shapes, and pore sizes. While this method can be easily used together with other production strategies to tune the final 3D structure, the approach also suffers from some important drawbacks. The long fabrication time, the lack of mechanical properties and the limited control over fiber orientation and diameter are some important issues, which should be investigated further to obtain better control over the produced nanofibrous TE scaffolds. 

### 2.2. Molecular Self-Assembly

Very recently, molecular self-assembly is gaining attention as a novel technique to prepare nanofibrous TE scaffolds [71,72,73,74]. In contrast to TIPS and electrospinning, which are top-down approaches, molecular self-assembly is a bottom-up method relying on the spontaneous arrangement of single macromolecules (basic units) into stable nanoscale supramolecular structures [75,76,77]. When appropriate stimuli are present, biomolecules have an inert tendency to self-assemble into supramolecular structures. Hydrophobic forces are the major driving forces for self-assembly and the self-assembled structures are typically stabilized by various attractive forces, like electrostatic interactions, hydrogen bonds, van der Waals forces [36,75,76,78]. As such, very stable molecular assemblies can be obtained despite the fact that all involved bonds are rather weak [36,78,79]. The structure of the self-assembled materials can be typically tuned by controlling the kinetics, the molecular chemistry and the assembly environment (for example pH, solvent, light, salt addition and temperature) [80]. However, the main difficulty in molecular assembly is to create molecular units that are able to trigger spontaneous arrangements into a precise structure, which mimics the nanostructured characteristics of the ECM [60].

**Figure 6:**
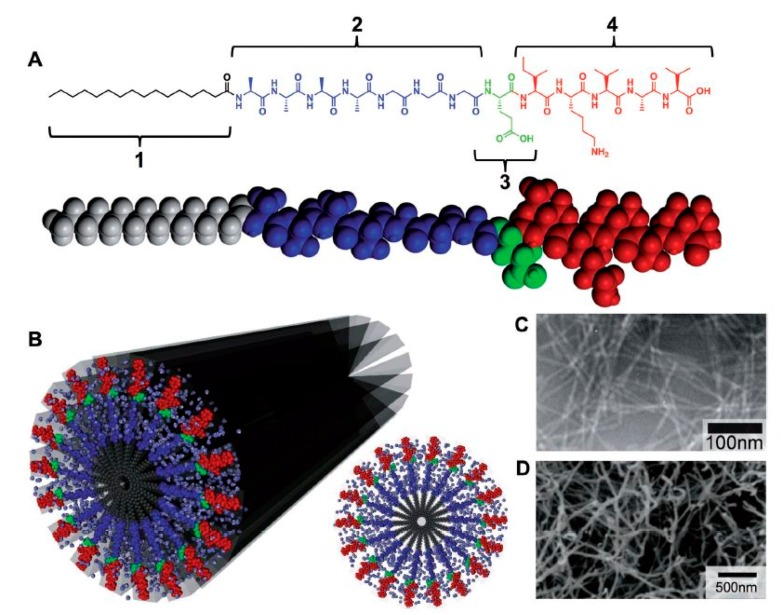
(**A**) Molecular structure of a peptide-amphiphile (PA) with four rationally designed chemical entities. (**B**) Molecular graphic illustration of the PA molecule and its self-assembly into nanofibers in addition to a schematic of the cross-section of these fibers (**C**) TEM images of isolucinelysine-valine-alanine-valine (IKVAV) nanofibers and (**D**) SEM micrograph of an IKVAV nanofiber mesh—reproduced with permission from [82]. Copyright Wiley, 2013.

Among different building blocks which can self-assemble into nanofibrous scaffolds (nucleic acids, proteins, and peptides), peptide-amphiphile (PA) blocks that integrate the activities of peptides with surfactant properties, have drawn a lot of attraction in TE applications as a result of their design flexibility and their ability to self-assemble in aqueous media [35,81]. The chemical design of a PA unit is typically composed of 4 main structural characteristics, as schematically depicted in Figure 6A. The hydrophobic tail is the first division which commonly consists of a long, saturated alkyl domain. Right next to the tail is the second section, which is made up of a small peptide sequence triggering hydrogen bonding by the creation of intermolecular β-sheets [82,83]. A third part is usually composed of acidic or basic amino acids to induce charge, promote water solubility and structural alternations (e.g., salt addition or gelation using a pH shift). The last segment, at the final point opposite the hydrophobic tail, is present to provide a bioactive signal to the unit and may be comprised of an epitope to engage with cell receptors, a section that immobilizes proteins or biomolecules or a pharmacological drug [82,83]. 

The main function of the hydrophobic tail, a crucial element in self-assembled systems, is strengthening the amphiphilic nature of the molecule. The amphiphilic nature of the molecule causes the alkyl moiety to collapse hydrophobically into the core of the nanostructure. As a result, the attached peptides are particularly placed on the surface of the nanofibers, where they can be easily accessed by cells, proteins or other biological targets. In aqueous solutions, the following three major forces are controlling the self-assembly of PA units: hydrophobic collapse of the alkyl tails, hydrogen interactions and side-chain bonding in the main peptide sections and electrostatic repulsions within the charged amino acids [82,83]. The interplay between all these interactions determine the ultimate architecture of the assembled PA units [78,81,82,83].

In 2002, Hartgerink et al. [84] showed that the self-assembly process into nanofibers can be influenced by tuning the hydrophobic tail. In a first step, these authors examined the influence of the hydrophobic tail length on the self-assembly process by varying the tail length from as long as a 22-carbon fatty acid to molecules containing no fatty acid at all. The authors observed that only the 10-, 16- and 22-carbon fatty acid were able to result in the formation of a dense network of fibers in excess of 1 µm long possessing fiber diameter in the range 5–8 nm [84]. The authors also observed that when the PAs with longer hydrophobic tails are oxidized, the self-assembly process into nanofibers is suppressed due to the formation of intramolecular disulfide bonds.

Peptide epitopes are another crucial element in self-assembling PAs and are located at the end of the molecule, opposite the hydrophobic tail. These epitopes enable the display of bioactive signals at controllable densities on the surface of the supramolecular nanofiber. For instance, in the case of interaction of cells with PAs, choosing epitopes that promote cell attachment is often favorable. RGDS, a short peptide epitope that is present in most of the ECM proteins, especially in fibronectin, is also known as a common epitope that improves initial cell attachment [82]. As a result, RGDS has been frequently incorporated into PAs to improve the biological response of the PAs [85,86]. Another commonly used epitope is the laminin-derived isolucinelysine-valine-alanine-valine (IKVAV), a pentapeptide sequence crucial for neuron cell adhesion, proliferation, migration and neurite growth [87,88]. Consequently, IKVAV has been often incorporated into PAs to improve their bioactivity in nerve TE applications [89,90,91]. A transmission electron microscopy (TEM) image of nanofibers, self-assembled from an IKVAV-terminated PA, can be found in Figure 6C showing several micrometers long nanofibers with nearly uniform fiber diameters of 7 ± 1 nm [81].

By controlling the process parameters, it is possible to tune the structural properties of the nanostructures obtained through PA molecular self-assembly. A widely used approach to tune the shape of the assembled nanostructures is by making alterations to the design of the β-sheet peptide segment. At first, it was found that modifications of this peptide part did not strongly affect the structure of the self-assembled PAs as in all cases cylindrical nanofibers were produced [83]. On the other hand, when sequences of alternating hydrophobic and hydrophilic amino acids were used, specific PAs were observed to self-assemble into completely flat nanobelts possessing a monodisperse width of 150 nm and lengths up to 0.1 mm [92]. In another study, Hung and Stupp generated bundles of aligned PA nanofibers by self-assembling PAs within parallel channels [93]. More information on other strategies used to tune the final morphology of PA nanostructures can be found in the review paper written by Webber et al. [82]. Researchers have also observed that 3D hydrogel networks, which mimic the highly hydrated nanofibrous structure of native ECM, can also be created from by the self-assembly of 1D PA nanofibers [82,94]. An example of such an IKVAV nanofiber gel network can be found in Figure 6C. 

To be applied in the field of TE, the self-assembled supramolecular structures should have a length scale more similar to natural tissues [94]. In an effort to obtain a higher length scale, some hierarchical supramolecular PA assemblies have been developed [95,96]. In a recent study, Capito and co-workers obtained a highly ordered architecture by adding drops of a negatively charged hyaluronic acid (HA) solution into a positively charged PA solution [95]. Upon contact of the two solutions, PA molecules promptly formed aligned bundles perpendicular to the diffusion barrier at the liquid–liquid interconnected layer, resulting in the production of a microsac-like mesh. This mesh could be penetrated by proteins and growth factors which in turn resulted in an excellent viability of encapsulated stem cells up to four weeks. Furthermore, when chondrogenic media were added, the cells even differentiated into chondrocytes. This unique microsac-like mesh could thus be potentially used in different regenerative medicine applications such as TE scaffolds or drug delivery vehicles. In another study, Zhang et al. [96] successfully fabricated massively aligned PA nanofiber bundles highly resembling the strongly aligned extracelullar fibrils in the brain, spinal cord and heart tissue. A simple heat and cool approach was used in their paper to obtain an aqueous liquid crystal solution: in a first step, the PA solution was dehydrated using heating after which a slow cooling process was conducted to transform the bundled fibers into a liquid crystalline solution. In the next step, this solution was added to a calcium chloride solution resulting in the fabrication of aligned fibers with macroscale length, which could potentially be used as artificial blood vessels. 

To conclude, it can be stated that molecular self-assembly is a relatively new approach to fabricate nanofibrous TE scaffolds. As a result, several technical hurdles still need to be addressed at this moment before the self-assembled nanofibrous structures can be successfully applied in the field of TE. For example, excellent control over pore size and pore structure has not yet been achieved although these are important parameters strongly affecting cell adhesion, proliferation, and migration. Moreover, there is no information on the degradation rate of these self-assembled nanostructures [97]. Finally, the mechanical properties of most self-assembled scaffolds are very poor and absolutely not sufficient to tolerate the mechanical forces coming from surrounding tissues.

### 2.3. Electrospinning

Electrospinning is an electrostatically driven process employed for the fabrication of inter-porous nanofibrous meshes from a huge range of materials such as polymers [98,99], inorganics and hybrid (organic–inorganic) compounds [100]. This nanofiber fabrication technique has been introduced in the 1930s, however, its applications were limited to filtration until the 1990s [101]. From that moment on, the technique also started to attract interest in other application fields due to the possible utilization of a wide variety of starting materials. Compared to the above-mentioned techniques, electrospinning is by far the most commonly used nanofibrous scaffold fabrication technique (see Figure 2) due to its cost-effectiveness [102], its simplicity [40], its immense versatility [103], its readiness for industrial scale-up and its large flexibility [104]. 

**Figure 7:**
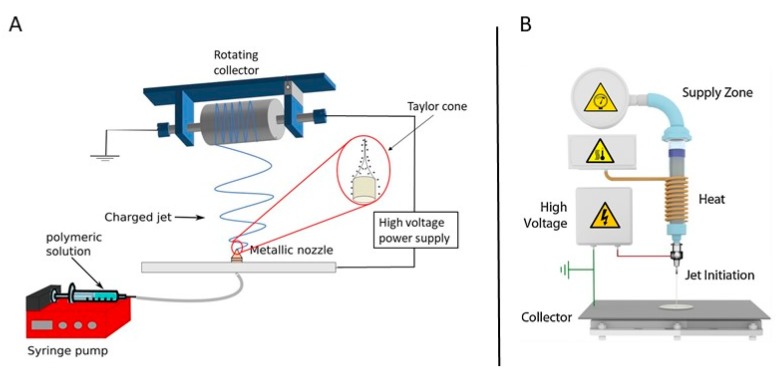
A schematic representation of (**A**) a polymer solution reproduced with permission from [106]. American chemical society, 2018 and (**B**) polymer melt electrospinning system—reproduced with permission from [107]. Copyright Elsevier, 2016.

In the field of TE engineering, most attention has been paid to the fabrication of electrospun polymer-based nanofibrous scaffolds and only these polymeric electrospun scaffolds will, therefore, be described in this paper. In this case, the formation of nanofibers is based on the uniaxial stretching of a viscoelastic liquid polymer solution (Figure 7A) or polymer melt (Figure 7B) using an electric field to eject a thin liquid jet from a needle or small capillary tube. Using this technique, nanofibers can be fabricated from a wide range of polymers or copolymers with diameters ranging from 3 nm up to 10 mm. Moreover, a variety of (macro) molecules can also be incorporated during the electrospinning process to produce highly-functionalized nanofibers [101,105] again evidencing the high flexibility of the electrospinning process.

A basic electrospinning set-up typically consists of three main parts: (1) a high-voltage direct current power source (with either negative or positive polarity), (2) a syringe pump or extruder/plunger feeding a polymer solution/polymer melt to a small orifice (a so-called spinneret) in case of solution and melt electrospinning respectively and (3) a collector. As previously mentioned, the principle of electrospinning is based on using an electric field to draw a polymer solution/melt from an orifice to a collector [98,108]. The mechanism behind this phenomenon is as follows: when a high voltage/low current is applied to the capillary tip, it polarizes the droplet of the polymer solution/melt at the tip of the capillary and induces electrical charges that will be accumulated over the droplet surface. However, when the electrostatic force overcomes the surface tension of the polymer liquid/melt, the polymer droplet is ejected, and a so-called Taylor cone is formed. The electrostatic repulsions between the surface charges of the droplet and the Coulomb forces caused by the strong externally applied electric field are the main reason for this deformation (conical shape). When the applied electric field exceeds a critical value, violent whipping motions of the polymer jet occur which in turn results in the formation of more elongating forces leading to faster evaporation of the solvent (or cooling of the polymer melt) to form solid randomly oriented polymeric nanofibers on the collector. The collector is usually constructed from electric conductors to neutralize the charge carried by the nanofibers [37,101,109,110]. As most attention in the field of TE has been paid to solution electrospinning, only this technique will be further elaborated in this review paper. More information on melt electrospinning and its application in TE can be found in an interesting review written by Muerza-Cascante et al. [111].

The physical properties of polymeric nanofibers fabricated by solution electrospinning can be controlled by a multitude of electrospinning parameters: (1) solution properties such as type of polymer, polymer concentration, polymer molecular weight, which in turn determine the solution viscosity, solution conductivity, and solution surface tension; (2) electrospinning process variables such as applied voltage, collector composition and geometry, capillary-to-collector distance, polymer solution feed rate and orifice size and (3) ambient parameters such as relative humidity, temperature and surrounded air velocity in the chamber [112]. As multiple review papers [37,113,114] have already described the effect of each of the mentioned parameters on the final physical characteristics of polymeric nanofibers, the reader is referred to these excellent papers to gather more information on this topic. Each of the above-mentioned parameters needs to be carefully controlled for each polymer/solvent combination to obtain nicely elongated, bead-free polymeric nanofibers.

**Figure 8:**
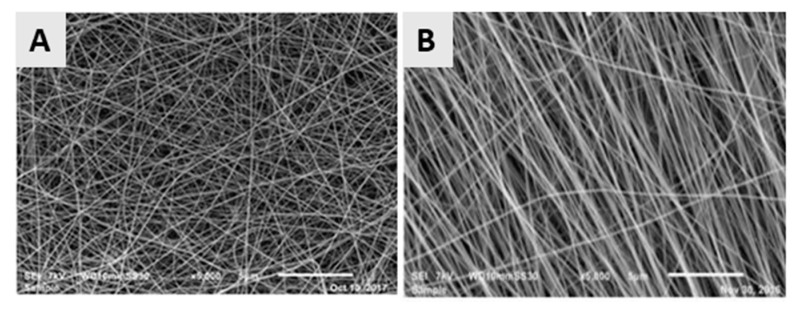
SEM images of the poly-ε-caprolactone (PCL) (**A**) Random nanofibers; collector speed: 300 rpm—Reproduced with permission from [106]. American Chemical society, 2018 and (**B**) aligned nanofibers; collector speed: 3000 rpm (for both images: concentration = 14%, mixture of formic and acetic acid (9:1), voltage: 32–33 kV)—reproduced with permission from [119]. Copyright Elseivier, 2018.

In a classical electrospinning set-up using a flat collector or a low-speed rotating cylindrical collector, the electrospun nanofibers are randomly arranged with interconnecting pores, as depicted in Figure 8A, which shows a PCL nanofibrous mat obtained from a 14% *w/v* PCL solution in a ratio of 9:1 formic acid/acetic acid mixture using a slowly rotating cylindrical collector. For some TE applications like nerve or tendon regeneration, it is however required to construct electrospun nanofibrous scaffolds consisting of aligned nanofibers, as the orientation of the nanofibers can have a positive influence on the mechanical properties of the scaffold and can aid to guide the orientation of cells seeded on these scaffolds [37]. For example, Chew et al. [115] and Wang et al. [116] reported the influence of aligned electrospun nanofibrous mats on the guidance of cultured cells and observed that nanofiber alignment can facilitate the elongation and orientation of cells along the alignment axis. Currently, a number of alternative electrospinning collectors have been developed to obtain aligned electrospun nanofibers [117,118], which can be categorized into 3 groups according to the type of forces used for fiber alignment: (1) a disc or mandrel with high-speed rotation (mechanical forces) [119]; (2) parallel electrodes (electrostatic forces) and (3) parallel permanent magnets (magnetic forces) [120]. More information regarding these special collectors can be found in the review paper of Toe et al. [117]. An example of aligned electrospun PCL nanofibers obtained from a 14% *w/v* PCL solution in a ratio of 9:1 formic acid/acetic acid mixture using a fast rotating disc (3000 rpm) can be observed in Figure 8B [119].

**Figure 9:**
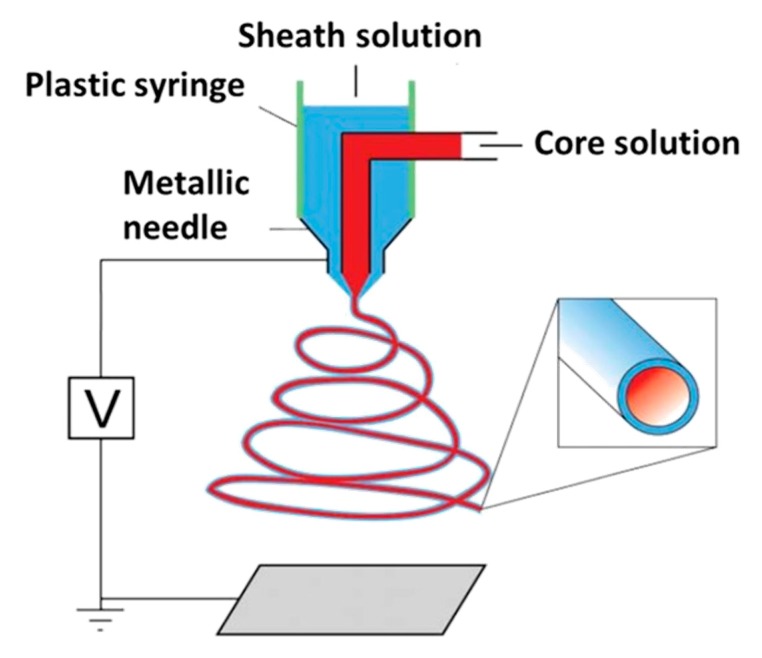
Schematic representation of an electrospinning set-up used for coaxial electrospinning—reproduced with permission from [131]. Copyright American Chemical Society, 2004.

Besides the conventional electrospinning process described above, co-axial spinning has also been developed to obtain core-shell nanofibers by designing novel coaxial spinnerets [121,122,123,124]. In this case, two different solutions can be injected into a single spinneret consisting of two different coaxial capillary channels, as schematically depicted in Figure 9. The polymer solution constituting the outer layer of the nanofibers (the so-called sheath polymer solution) typically carries the charge and its conical shape at the tip of the needle/capillary causes the core layer formed by the so-called core polymer solution to deform. As a consequence, using this technique, some difficult–to–spin polymers can also be electrospun as they can form an ultrafine core inside the shell of an easily spinnable polymer. In a later stage, the polymer shell can be dissolved/removed, thereby revealing the spin-target polymer nanofiber. A similar concept can also be used to remove the core of the resultant nanofibers to fabricate hollow nanofibers by choosing a core polymer solution which can be post-spinning dissolved in a particular solvent and by selecting a sheath layer which is insoluble in the same solvent. Currently, there are different studies available focusing on the fabrication of core-sheath and hollow polymeric nanofibers [125,126,127]. Inside hollow polymeric nanofibers, biologically active agents (for example, drugs) can be encapsulated to form a drug delivery device [121]. Core–sheath nanofibers are also very interesting for TE applications as they can facilitate tissue regeneration processes by the incorporation of biomolecules (for example peptide or growth factors) into the core of core-shell nanofibers [128,129]. Additionally, by adding different components to the core and shell of core-shell nanofibers, the simultaneous release of these components during the tissue regeneration process can be achieved [130]. Compared to single component nanofibers, these hollow and coaxial nanofibers can have several benefits in biomedical applications: (1) bioactive agents present in the core are not exposed to a harsh chemical environment as they are shielded by the surrounding shell part; (2) by carefully controlling the thickness of the core and shell layer, a well-controlled release of a drug can be obtained; (3) by selecting two suitable compounds, the coaxial nanofibers can have excellent mechanical properties and (4) a polymer with a higher biocompatibility can be chosen as sheath material while a polymer with lower biocompatibility can be used as a core [120].

**Figure 10:**
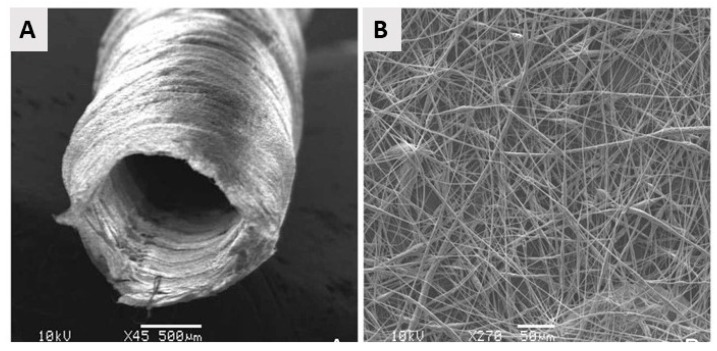
SEM images of the electrospun poly(lactic-co-glycolic acid) (PLGA)/PCL nerve guide conduit and (**A**) magnified details of the tube wall (**B**): microfibers and nanofibers range in diameter from approximately 280 nm to 8 μm—reproduced with permission from [138]. Copyright BMC biotechnology, 2008.

From the above, it can be concluded that by using electrospinning nanofibrous meshes with interconnected pores can be produced, which are strongly resembling the natural ECM. As such, these electrospun meshes can potentially be used as scaffolds in the field of TE. Unfortunately, the nanofibrous meshes fabricated through conventional electrospinning are usually pseudo 2D rather than 3D, thereby preventing the required cell diffusion for TE applications. To deal with this issue, several approaches to form 3D electrospun scaffolds have already been developed. A commonly applied technique is using a liquid reservoir as a collector during the electrospinning process: when low surface tension liquids such as methanol, ethanol, and water are used, the electrospun nanofibers sink during electrospinning process minimizing fiber bonding [132]. This results in the fabrication of a fluffy layer of nanofibrous meshes with higher internal porosity [133]. Tubular electrospun nanofibrous conduits can also be produced by depositing fibers on a small diameter cylindrical collector [134]. These tubular conduits are commonly used in nerve or vascular TE as they are able to resemble the structure of these tissues [135,136,137]. For example, electrospun nanofiber conduits have been fabricated with a length of 10 mm and a total wall thickness of 155 µm by electrospinning a mixture of PCL and PLGA on a rotating rod possessing a diameter of 1.29 mm [138]. In a first step, pure PCL nanofibers were electrospun with an average diameter of 7.5 ± 2.0 µm, followed by the electrospinning of a mixture of PCL and PLGA resulting in the deposition of PCL/PLGA nanofibers with an average fiber diameter of 279 ± 87 nm. An SEM image of the obtained electrospun conduit can be seen in Figure 10A, while an SEM image of the conduit wall can be observed in Figure 10B. Additional information regarding the formation of 3D electrospun scaffolds can be found in a recent review paper written by Jun et al. [132].

Currently, significant attention is being paid to the up-scaling of the electrospinning process to enable mass production of electrospun nanofibrous mats. In a laboratory setting, researchers are mostly using polymer volumes in the range of milliliters although it is possible to electrospin several liters of a polymer solution in a continuous run using a lab-scaled electrospinning process. The most important limitations to up-scale the electrospinning process are mentioned hereafter: (1) for most materials, the maximal attainable length range of electrospun nanofibers is ranging from hundreds of micrometers to millimeters. As such, electrospinning presents poor reproducibility when applying it on a commercial scale. (2) Optimization of the electrospinning process is not easy due to the weak viscoelastic character of most polymer solutions, the poor solubility of polymers into different solvents, the lack of adequate molecular entanglements, and, more generally, because only some of the electrospinning processing parameters can be easily varied. Up to now, several researchers are however trying to increase the productivity of the electrospinning process as comprehensively discussed in the review paper of Persano et al. [104]. The most relevant technological improvements that have, to date, been investigated to promote the mass production of electrospun mats are mainly based on the modification on modifying the system of polymer injection and consist of: (1) the use of multi-spinnerets that allow parallel multi-processing [139] and (2) the introduction of free surface or so-called needleless electrospinning methods [140], such as for example bubble electrospinning [141] and roller electrospinning [142]. More in-depth knowledge of multi-needle and needleless electrospinning can be found in the review papers written by Yu et al. [143] and Persano et al. [104].

### 2.4. Comparison of Different Nanofibrous Scaffold Fabrication Techniques

To conclude the part on the fabrication of nanofibrous TE scaffolds, it can be stated that selecting the most suited method for nanofiber fabrication highly depends on two main factors: (1) the envisioned starting materials as the used technique should not damage or denature these materials during the nanofiber fabrication process when using solvents, high voltage, heating, etc., and (2) the foreseen application which typically specifies the required nanofiber morphology like fiber diameter, porosity, fiber direction, required fiber alignment and fiber length. A general overview of the advantages and disadvantages of each described nanofiber fabrication technique can be found in Table 1, helping the reader to obtain a general overview of the pros and cons of each method. In the next section, the most widely applied synthetic polymers used to generate electrospun nanofibrous scaffolds will be briefly discussed to help researchers choosing the most appropriate synthetic polymer for a practical envisioned TE application.

## 3. Synthetic Biodegradable Polyesters Used to Fabricate Electrospun TE Scaffolds

**Figure 11:**
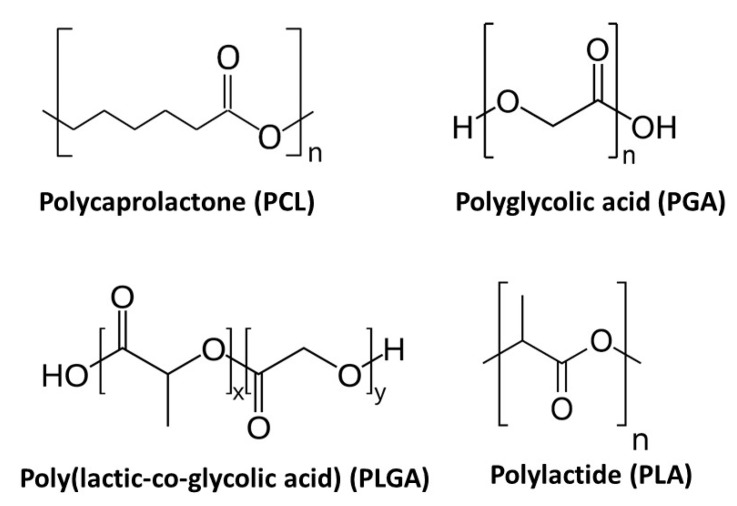
Chemical structure of the most commonly used synthetic polymers for the fabrication of electrospun TE scaffolds.

As mentioned in the previous section, of all the current strategies available for synthesizing polymeric nanofibers, electrospinning is considered the most straightforward and cost-effective technique. Although a large variety of natural and synthetic polymers has already been successfully electrospun into TE scaffolds, this section will only focus on biodegradable aliphatic polyesters as these polymeric materials are the most commonly used for the fabrication of nanofibrous TE scaffolds using solvent electrospinning [144]. Their success in the biomedical field is based on their excellent mechanical properties, their high biocompatibility and their intrinsic biodegradable properties [145]. Indeed, when incubated in aqueous solutions, aliphatic polyesters undergo hydrolytic degradation of the present ester linkages. This degradation process strongly depends on the length of the alkane backbone and complete degradation of aliphatic polyesters typically occurs in a timeframe of a few months up to several years, depending on their chemical structure [146,147,148]. Due to their relatively slow degradation process, aliphatic polyesters are mainly investigated as a material for long-term TE applications [147,148]. In the following, the most important aliphatic polyesters (chemical structures shown in Figure 11) will be shortly described. Additionally, the fabrication of electrospun nanofibrous scaffolds from these polyesters will also be reviewed.

### 3.1. Polyglycolic Acid

Polyglycolic acid (PGA) or polyglycolide is the simplest linear aliphatic polyester and is a rigid thermoplastic polymer. PGA is a crystalline polymer with a high degree of crystallinity (44–55%) and consequently shows great mechanical properties. At the same time, it also shows low solubility in most organic solvents, making it difficult to use PGA for solvent electrospinning [149,150,151,152]. PGA is however soluble in highly-fluorinated solvents, such as for example hexafluoroisopropanol (HFIP). The PGA degradation procedure commonly takes place in two steps: firstly, hydrolytic chain scission of the ester group occurs as a result of water diffusion into the amorphous regions of the polymer. Secondly, after erosion of the amorphous parts, the hydrolytic attack of the crystalline parts of the polymer starts to occur. PGA loses its strength within 1–2 months after initiation of the hydrolysis process and loses its mass over 6 to 12 months. In vivo, glycolic acid is produced as a result of PGA degradation. This degradation product is non-toxic and can be either removed from the body through the urine or converted into CO_2_ and water which can be excreted via the respiratory system [150,151,153].

**Figure 12:**
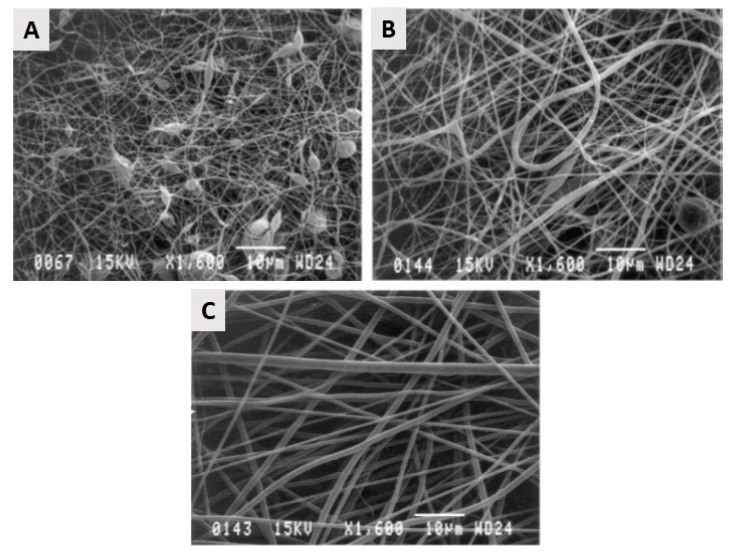
Scanning electron micrographs of control-group electrospun PGA at 1600× magnification. (**A**): 67 mg/mL with 0.22 ± 0.07-µm fibers (excluding beads) and 1.84 ± 1.08-µm^2^ pores. (**B**): 100 mg/mL with 0.42 ± 0.17-µm fibers and 3.53 ± 2.78-µm^2^ pores. (**C**): 143 mg/mL with 0.88 ± 0.37-µm fibers and 13.22 ± 7.45-µm^2^ pores—reproduced with permission from [155]. Copyright Wiley, 2004.

PGA has already been successfully electrospun starting from a PGA solution in HFIP with PGA concentration varying between 5 and 14 *w/v*%. Over the examined PGA concentration range, electrospun PGA nanofibers were generated with average fiber diameters ranging from 110 nm to 1.19 µm with higher fiber diameters obtained at higher PGA concentrations. Using these PGA solutions, nicely elongated electrospun PGA nanofibers were only obtained at PGA concentrations above 10 *w/v*%, while at lower PGA concentrations, beads were found to be present in the PGA electrospun scaffolds [154,155], as shown in Figure 12. The high degradation rate, as well as the limited solubility of PGA, do limit the applicability of PGA nanofibrous TE scaffolds. Therefore, several co-polymers of PGA have been introduced to prevail the inherent shortcomings of PGA, of which Poly (PLGA) is the by far the most applied co-polymer, as will be discussed later on.

### 3.2. Polylactic Acid

A second, very important biodegradable linear aliphatic polyester is polylactic acid (PLA), which is known as one of the most promising biopolymers for TE applications [156]. Due to the chiral structure of lactic acid, there are different types of PLA. The most commonly used ones are PLLA poly (l-lactic acid) and poly (dl-lactic acid) (PDLA). Similar to PGA, PLLA is a crystalline polymer possessing roughly 37% of crystallinity depending on molecular weight and the conditions of polymer processing. Compared to PGA, PLLA is also a more slowly-degrading polymer: when being hydrolyzed, the polymer loses its strength in almost 6 months. However, no remarkable alternation in mass occurs for a long term and complete resorption of high molecular weight PLLA can occur only after several years. In addition to the low degradation rate, PLLA also has excellent tensile strength, a high tensile modulus, and a low elongation at break. In contrast, PDLA is an amorphous polymer which consequently shows a smaller strength value than PLLA. Additionally, PDLA also degrades faster than PLLA: it loses its strength in 1 to 2 months as a result of hydrolysis and loses mass within 12 to 16 months. In vivo, PLA is hydrolyzed into its repeating unit, lactic acid. This chemical compound is a normal human metabolic by-product and is in vivo degraded to water and CO_2_, which can be subsequently removed by the human respiratory system. Due to their excellent solubility in different solvents, PLLA and PDLA have been successfully electrospun starting from different polymer/solvent systems, although mostly chlorinated and fluorinated solvents have been used. An overview of some examples of the fabrication of electrospun PLLA and PDLA scaffolds can be found in Table 2 and Table 3 respectively combined with the applied electrospinning parameters and the obtained nanofiber characteristics.

### 3.3. Poly-ε-Caprolactone

One of the most widely applied aliphatic polyesters to fabricate electrospun TE scaffolds is PCL. PCL is a semi-crystalline synthetic polymer in which has a very low glass-transition temperature (approximately −62 °C), making it highly elastic at room temperature. PCL also has a low melting point of 55–60 °C, depending on the degree of crystallinity, which is in turn dictated by the molecular weight and the scaffold fabrication process. Additionally, PCL is readily soluble in a wide range of organic solvents, making it an easy-to-electrospin polymer. Due to its semi-crystalline and hydrophobic nature, PCL exhibits a very slow degradation rate (2–4 years depending on the starting molecular weight) and has mechanical properties suitable for TE applications [166]. Like the earlier discussed aliphatic polyesters, PCL also hydrolytically degrades owing to the presence of ester linkages. Some examples of the successful fabrication of electrospun PCL nanofibrous scaffolds for TE applications can be found in Table 4. This table clearly reveals that the use of chlorinated solvents results in the fabrication of rather thick PCL nanofibers (fiber diameter >400 nm), while the use of the solvent mixture formic acid/acetic acid results in very thin PCL nanofibers possessing an average fiber diameter close to 100 nm.

### 3.4. Poly (Lactic-Co-Glycolic acid)

In an effort to tune the mechanical properties and the degradation kinetics of aliphatic biodegradable polyesters, different co-polyesters have also been developed, of which the amorphous PLGA is by far the most widely applied as starting material for TE scaffolds. In contrary to the homo-polymers of lactic acid and glycolic acid which show a rather poor solubility, PLGA is easily soluble in a wide variety of solvents. PLGA also degrades through ester bond hydrolysis and the degradation rate of PLGA can be carefully controlled by changing the lactic acid/glycolic acid ratio. For example, PLGA with a PLA: PGA ratio of 85:15 degrades in 5–6 months, while PLGA with a PLA: PGA ratio of 50:50 degrades in 1 to 2 months. In fact, increasing the percentage of glycolic acid with respect to lactic acid in the copolymer generally increases the rate of degradation both in vitro and in vivo [173]. PLGA nanofibers with varying lactic acid/glycolic acid ratios have been successfully fabricated making use of solvent electrospinning. Some selected examples of PLGA electrospinning are presented in Table 5 in combination with the used electrospinning parameters and the obtained PLGA nanofiber morphology.

### 3.5. Other Co-Polyesters

Besides PLGA, also other co-polyesters such as PCL/PLA and PCL/PGA have been investigated as possible TE scaffolds in an effort to further tune the bulk properties of TE scaffolds. In a recent publication, Yao et al. [176] have compared the performance of 3D electrospun PCL and PCL/PLA (mass ratio: 4/1) blend nanofibrous scaffolds in bone TE applications. The observed that compared to the pure PCL scaffolds, PCL/PLA blend scaffolds had better mechanical properties and in vitro bioactivity. Consequently, the blended scaffolds not only enhanced the cell viability of human mesenchymal stem cells (hMSCs), but also promoted the osteogenic differentiation. Furthermore, in vivo studies also revealed that the PCL/PLA scaffolds considerably facilitated new bone formation in a critical-sized cranial bone defect mouse model. Blended PCL/PLA nanofibrous scaffolds can thus have very important applications in bone TE. In another paper authored by Aghdami et al. [177], randomly oriented nanofibrous meshes made of both PCL and PGA with various PCL/PGA compositions (100/0, 80/20, 65/35, 50/50, and 0/100) were produced using solvent electrospinning. Morphological characterization of the obtained nanofibers revealed that the nanofibers’ average diameters increased when increasing amounts of PGA were added to PCL. Additionally, the wettability of the nanofibrous meshes also increased when the PGA amount in the solution mixture was increased. Finally, the mechanical characterization of the nanofibrous meshes also revealed that enhancing the amount of PGA resulted in a tremendous increase in the mechanical properties. By changing the PCL/PGA ratio, the morphological, chemical and mechanical properties of the TE scaffold can thus be fine-tuned to meet the requirements for a particular TE application. Besides the blending of different biodegradable polyesters, these polyesters can also be mixed with natural polymers for TE applications to improve specific nanofiber characteristics such as mechanical strength, elasticity, wettability and/or electrospinnability [173]. These blends will however not be described in this particular review paper as numerous information regarding this topic can already be found in the very interesting review paper of Sell et al. [178].

Although a wide range of synthetic biodegradable polyesters are utilized as electrospun TE scaffolds, a major disadvantage of this class of materials is a lack of biological recognition on their surface as a result of their inherent biological inert properties [179]. Indeed, surface biochemical cues can significantly affect cell-surface behavior as they are initiator/elicitor for most of the biological responses [180]. As such, the scaffold surface often needs to be modified to obtain more desirable characteristics to promote cell-scaffold interactions. Despite different surface modification techniques are available for this purpose as thoroughly reviewed in [80], this paper will particularly focus on non-thermal plasma (NTP) surface modification, which has proven to be a very promising technique for scaffold surface modification.

## 4. Plasma-Assisted Surface Modification of TE Scaffolds

As already mentioned, biodegradable aliphatic polyesters have been comprehensively investigated as electrospun TE scaffolds as they show low immunogenicity, excellent mechanical properties, non-toxic behavior, and controllable degradation rates. However, the hydrophobic nature and low surface energy of these polymers often result in poor cell attachment. Consequently, modification of the surface of TE scaffolds fabricated from synthetic polyesters is commonly required and various strategies have already been developed to tackle this major drawback [144,181,182,183]. A commonly used means of surface functionalization is through wet chemistry, which relies on the immersion of the nanofibrous scaffolds into harsh chemicals, such as strong acids or alkalis (for example H_2_SO_4_ or NaOH) [184,185]. This strategy enables the grafting of hydroxyl or carboxylic groups by hydrolysis through nucleophilic acyl substitution [186]. The grafting of these oxygen-rich groups was found to improve the hydrophilicity and initial cell attachment [185,187]. Alternatively, amino groups can also be grafted onto the surface of TE scaffolds via aminolysis, a process in which the nanofibers are immersed in, for example, 1,6-hexanediamine [188]. Unfortunately, due to the harsh processing conditions, the bulk physical properties easily deteriorate due to the scission of the polymer backbone [184,189]. Additionally, these chemical processes are usually time-consuming and generate lots of liquid waste which should be disposed of carefully. Moreover, there is the risk of inadequate washing, which could result in the presence of residual chemicals within the nanofibrous matrix, thereby severely compromising the biocompatibility of the TE scaffold.

Compared to the wet chemical route, NTP-assisted surface modification of TE scaffolds is considered to be a much more suitable technique to improve cellular interactions on TE scaffolds. This is attributed to their numerous advantages, of which a few will be mentioned hereafter. Plasma treatments are performed in the gaseous phase and do not use any harsh chemical, making them a greener alternative. In addition, plasma effects are usually limited to the first few subsurface layers, not influencing the bulk properties of TE scaffolds. On top of that, as no solvents are used, there is a minimal risk of chemical contamination afterwards. Moreover, under the right plasma conditions, scaffolds will only experience minimum heating, thus avoiding thermal damage to the delicate nanofibrous structures [190]. Due to the mentioned benefits, plasma surface treatments of TE scaffolds show high potential to enhance cell-scaffold interactions and will, therefore, be the main subject of the following sections. First, a general introduction to plasma and different plasma set-ups will be given, followed by a comprehensive overview of recent findings on plasma treatments of nanofibrous TE scaffolds.

### 4.1. Plasma Set-Ups Used for the Treatment of Nanofibrous TE Scaffolds

In physics, plasma is defined as an ionized gas consisting of positive and negative charges in equal density and is also known as the fourth state of matter. Plasma can be categorized into 2 different categories (thermal and non-thermal plasma), depending on its thermal equilibrium state. In the case of a thermal plasma, also known as a hot or equilibrium plasma, the electron and ion temperature are in equilibrium. Due to the high ion temperature, the overall gas temperature is typically several 1000 K and this plasma type is therefore not considered suitable for the treatment of heat-sensitive materials such as TE scaffolds. Equilibrium plasmas are mainly applied for the surface modification of metallic and silicon-wafer surfaces which can withstand the high operating gas temperatures to deposit a thin diamond-like coating, zinc oxide coatings and silicone films [191,192,193,194]. Other application fields can be found in the field of nanoparticle production and the destruction of hazardous waste [195].

However, in an NTP, also known as a non-equilibrium or cold plasma, the temperatures of electrons and ions/heavy particles are not in equilibrium. This stems from the methodology in which the plasma is generated: by using a strong electric or magnetic field, the free electrons are accelerated, resulting in ionization. Consequently, the relative velocity of the ions is much lower than the velocity of the electrons. This results in the formation of a plasma state at significantly lower gas temperatures, making these plasmas ideal for the modification of thermosensitive materials such as polymeric nanofibrous TE scaffolds [196]. A wide range of methods is currently available to generate NTPs, both at vacuum as well as atmospheric pressure. In this work, however, we will limit ourselves to the 2 plasma types which are mostly used for the treatment of nanofibrous TE scaffolds: (1) dielectric barrier discharges (DBDs) [197,198,199,200] and (2) radiofrequency (RF) discharges [201,202,203,204,205,206,207,208,209,210,211]. For more information on other available NTP set-ups, the reader is referred to extended review papers, focusing on the generation of NTPs [212,213,214,215].

**Figure 13:**
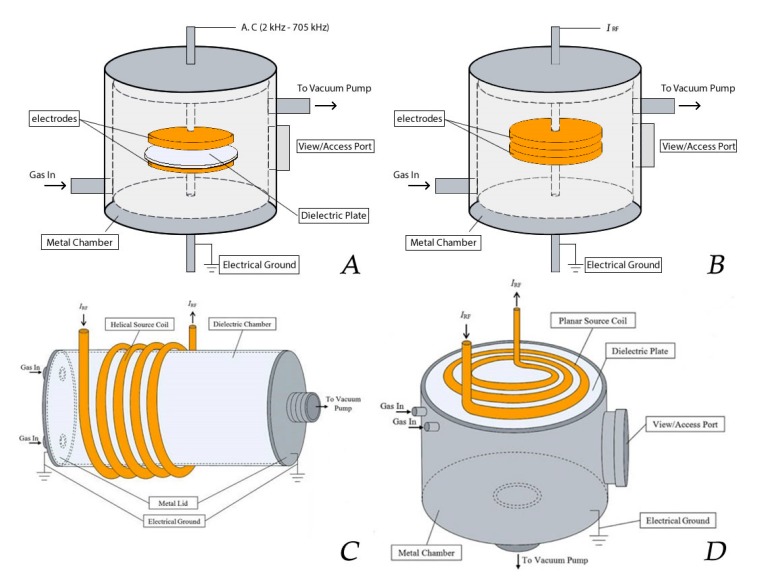
Typical plasma reactor set-ups of (**A**) a dielectric barrier discharges (DBD), (**B**) a capacitively coupled plasma (CCP) and (**C**) an inductively coupled plasma (ICP) with helical coil configuration and (**D**) an ICP with planar coil configuration—reproduced with permission from [230]. Copyright Springer, 2017.

One of the most popular NTP sources used for polymer surface treatment is the DBD. A DBD set-up applied for surface treatment typically consists of two parallel electrodes separated by a small gas gap of a few millimeters up to a few centimeters. Characteristics of a DBD are that at least one electrode is covered with a dielectric layer such as glass, quartz or alumina (see Figure 13A). When an AC high voltage with a frequency (typically in the range of kHz) is applied, the high intensity of the electrical field is created between the electrodes [190]. However, also other driving modes can be used with this plasma reactor type generating plasma discharges with characteristics different from DBDs [216]. Due to the presence of the dielectric layer(s), the charge is homogeneously distributed across the electrodes, encouraging the formation of micro-discharges rather than a single arc discharge. The ionization of the gas occurs near these micro-discharges due to the acceleration of the electrons from the cathode to the anode by the generation of a strong electric field [217]. Collisions between the accelerating electrons and the supplied gas cause the gas to be ionized, thereby generating and sustaining the plasma [190,218,219]. DBDs can be easily generated at atmospheric pressure [220,221,222,223,224,225,226], but also DBDs operating at medium pressure (1–10 kPa) are commonly applied for polymer surface modification [223,224,225].

Besides DBDs, also RF discharges are widely applied for plasma-assisted surface modification. RF discharges can be divided into two categories, capacitively coupled plasma (CCP) and inductively coupled plasma (ICP), depending on the method of RF power coupling [226]. A CCP, which is mainly generated at low pressure (10^−3^–1000 Pa), consists of the same electrode configuration as the one employed for DBD reactors (2 horizontal or vertical parallel plate electrodes), but without the presence of a dielectric [212,227]. This plasma type is mostly generated under vacuum conditions to maintain a stable glow discharge, as higher pressures would lead to instabilities that could transition the plasma from a (pseudo)glow mode to an arc discharge. Although CCPs can be driven by RF high voltage sources operating between 1 and 100 MHz, a high voltage power source operating at 13.56 MHz is by far the most used. The mechanism of ionization to generate a CCP is similar to the case of a DBD [228,229], however, micro-discharges are not generated because the electrons and ions in the plasma do not reach the electrodes due to the high operating frequency [212,227]. In the case of an ICP, the plasma is not generated by oscillating electrical fields, but by oscillating magnetic fields. These, in turn, induce a strong RF electric field in the plasma region, thereby accelerating the free electrons. Consequently, this causes the gas to be ionized, thereby generating and sustaining the plasma phase [212]. These discharges can be sustained without the need for the electrodes to be in contact with the plasma, which can be a huge benefit for different applications as in this way impurities originating from the electrodes can be prevented [230]. Although the discharges can be applied at high pressure and even at atmospheric pressure, in most instances the discharges are operated under vacuum. Inductive coupling is typically realized by wrapping an induction coil around a dielectric plasma chamber (helical coil configuration), which is in turn powered by an RF power source operating at 13.56 MHz. (Figure 13C). More recent designs employ a planar coil configuration, which consists of a flat spiral coil buffered by a dielectric plate, which is mounted onto a metal plasma chamber at vacuum pressure containing a substrate holder (see Figure 13D). In the latter case, the ICP is generated close to the plate and across the coil radius through a circular magnetic field. The advantage of the planar coil configuration is a more uniform density distribution of the ICP created across the coil dimensions, providing better procedure controllability and reproducibility of plasma surface treatments (see Table 6) [226,231].

When a material is exposed to one of the abovementioned plasma discharges, various plasma-surface interactions are possible. Three major types of plasma reactions can be recognized based on the outcome of the interaction: (1) plasma activation, (2) plasma polymerization and (3) plasma etching. As the third interaction as such is not employed on nanofibrous TE scaffolds, only the first two categories will be described in detail in this review paper. After a brief introduction of each technique, specific examples on nanofibrous TE scaffolds will be presented.

### 4.2. Plasma Activation

When the surface of a nanofibrous TE scaffold is exposed to a plasma generated in an inert atmosphere (such as helium (He) or argon (Ar)), active plasma species such as ions, electrons, and free radicals present within the plasma will bombard the scaffold surface. This process, also known as plasma activation, causes bond cleavage of the polymeric chains, thereby creating a surface rich in highly reactive free radicals. After removing the sample from the plasma reactor and exposing it to ambient air, these free surface radicals will be rapidly quenched by oxygen to form different oxygen-rich surface functional groups such as for example peroxides and hydroperoxides [232,233]. Oxygen or nitrogen functionalities can also be directly grafted on a surface using reactive instead of inert discharge gasses such as for example CO_2_, O_2_, air, N_2_ or NH_3_ [205,207,234,235]. In this case, the plasma also leads to the creation of surface radicals. However, these radicals rapidly react with readily available active plasma species resulting in the direct grafting of oxygen and/or nitrogen surface functional groups. As these incorporated functional groups are polar in nature, plasma activation is often applied to significantly increase the hydrophilicity of a surface, a process which is known to strongly improve the biocompatibility of otherwise inert materials [205,236,237,238].

Besides envisioning an increase in surface hydrophilicity, NTPs can also be employed to make surfaces more hydrophobic. This effect can be obtained when using fluorine-containing discharge gases such as for example CF_4_ or SF_6_ [239,240,241]. In this case, non-polar fluorine-containing surface functional groups are directly grafted on a surface, thereby significantly increasing the surface hydrophobicity. Although plasma activation processes are considered to be non-invasive, the higher-energy particles may lead to minor etching of nanofibrous TE scaffolds, thereby degrading their mechanical properties. As such, considerable attention needs to be given to the use of appropriate discharge powers and/or plasma exposure times [242,243].

In 2009, Martins et al. exposed electrospun PCL scaffolds to an RF discharge (type CCP) sustained in O_2_ and Ar using two different discharge powers (20 and 30 W) and varying plasma treatment times (5 and 10 min) [243]. A strong increase in surface hydrophilicity was observed for the O_2_ plasma-treated

**Figure 14:**
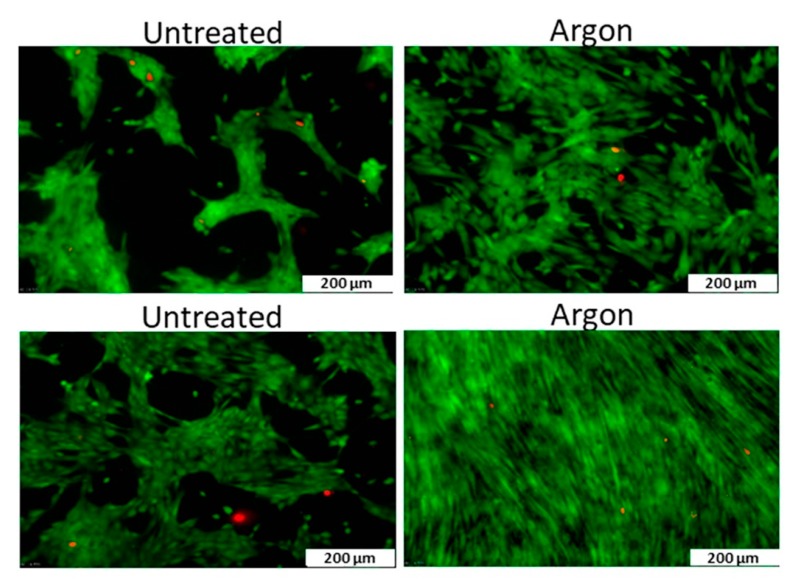
Fluorescent images one day (**top**) and seven days (**bottom**) post-seeding of human foreskin fibroblasts (HFF) cells after culturing on untreated and Ar plasma-modified PCL nanofibrous meshes—reproduced with permission from [106]. Copyright American Chemical Society, 2018.

PCL scaffolds as the water contact angle (WCA) values were found to decrease from 130° to values below 20°. Considering the WCA values of Ar plasma-treated PCL scaffolds, only a decrease of 20° in WCA value was observed, which means that the surface only became slightly less hydrophobic using this discharge gas. The elemental composition of the surface of the plasma-treated biomaterials was also characterized using X-ray photoelectron spectroscopy (XPS). The surface oxygen content on the O_2_ plasma-treated samples was found to increase with plasma exposure time and with applied discharge power, however, the combination of the highest discharge power (30 W) with the highest plasma treatment time (10 min) resulted in a decrease in surface oxygen content, most likely due to melting of the nanofibers initiated by these very harsh plasma conditions. In case of Ar plasma treatment, an increase in oxygen content was also observed with increasing plasma treatment time using a discharge power of 20 W. In contrast, a discharge power of 30 W resulted in a decrease in oxygen content compared to the untreated PCL sample, which was again caused by fiber damaging. These results thus clearly show that plasma operating parameters need to be carefully controlled to avoid damaging of fragile nanofibers. In a next step, Martins et al. examined the cellular interactions on all plasma-treated PCL scaffolds under study and observed that it was possible to define plasma treatment conditions leading to enhanced adhesion and proliferation of fibroblasts, chondrocytes and osteoblasts compared to the untreated electrospun PCL scaffold (O_2_ at 30 W for 5 min and Ar at 20 W for 5 min) [243]. O_2_ and Ar plasma treatments have also been conducted by Ivanova et al. [244] on electrospun PCL scaffolds using a low-pressure RF discharge at 30 W for 2 min. These authors also observed a strong decrease in WCA values, although some changes compared to the work of Martins et al. could be noticed. In the work of Ivanova et al. [244], the Ar plasma treatment was found to decrease the WCA by over 50°, while in the case of O_2_ plasma treatments, the WCA value was observed to decrease with more than 90°. The authors also observed a strong scaffold surface oxidation resulting from both Ar as well as O_2_ plasma treatments and suggested that in case of Ar plasma this oxidation may be originating from the presence of residual air in the plasma chamber or from the interaction of scaffold surface radicals with ambient air. Very recently, Asadian et al. used a medium pressure DBD sustained in Ar to modify the surface properties of electrospun PCL nanofibrous meshes [180]. A low discharge power of 1.8 W was used with treatment times up to 70 s. Similar observations as previously mentioned were done: Ar plasma treatment resulted in a strongly increased surface hydrophilicity due to the incorporation of oxygen-containing functional groups. SEM images also revealed that the morphology and the surface roughness of the PCL nanofibers remained unaffected by the conducted Ar plasma treatment, resulting from the mild plasma conditions. Additionally, the Ar plasma treatment was found to strongly enhance the adhesion and proliferation of human foreskin fibroblasts (HFFs), as evidenced by the fluorescent images taken one day and seven days post-seeding shown in Figure 14.

**Figure 15:**
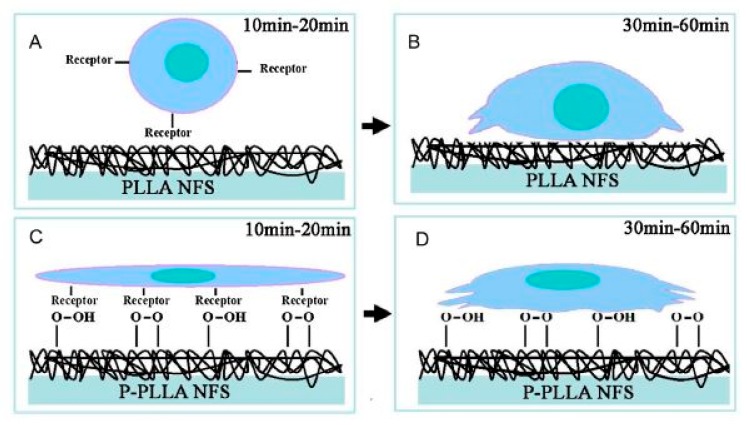
Schematic diagram of porcine mesenchymal stem cells (pMSCs) adhesion on pristine PLLA nanofibrous scaffolds (PLLA NFS) and O_2_ plasma-treated PLLA nanofibrous scaffolds (P-PLLA NFS)—reproduced with permission from [246]. Copyright Elsevier, 2014.

Next to PCL, also nanofibrous PLLA scaffolds have been functionalized using an O_2_ plasma. In 2014, Liu et al. used a low-pressure RF discharge to treat PLLA scaffolds, unfortunately, the used discharge power, as well as the applied plasma exposure time, were not mentioned in their publication [245]. It was found that the hydrophilicity of the PLLA nanofibers was strongly improved as the WCA value decreased from approximately 128° to 48°. Again, this enhanced wettability was attributed to the incorporation of oxygen-containing groups on the PLLA surfaces. The authors also investigated the impact of the performed plasma treatment on initial cell adhesion using porcine mesenchymal stem cells (pMSCs) and observed significantly enhanced cell adhesion on the plasma-modified PLLA sample. Moreover, results also revealed that plasma modification significantly affected the morphology of pMSCs, as schematically shown in Figure 15: in the case of pristine PLLA samples, pMSCs showed a round-shaped morphology 10 and 20 min post-seeding, while 30 to 60 min after seeding, the pMSCs started to slightly stretch. In contrast, on the plasma-treated PLLA scaffolds, a 2D planar morphology of the pMSCs was observed 10 and 20 min post-seeding. In addition, 30 to 60 min after seeding, the pMSCs were found to exhibit a 3D stretched morphology. These observations can be explained as follows: due to the high hydrophobic nature and lack of bioactive groups on the surface of pristine PLLA, it is hard for integrin receptors to find required binding sites to adhere to during the initial attachment procedure. Consequently, a ball-shaped cell morphology is observed at early time intervals (Figure 15A). After 20 min of culturing, focal adhesion occurred resulting only in very slight stretching of the cells on the nanofibrous PLLA surface (Figure 15B). As previously mentioned, O_2_ plasma modification resulted in the incorporation of oxygen-containing polar groups at the PLLA surface, which in turn improved the wettability of the PLLA nanofibers and supplied sufficient specific binding groups for integrin receptors to adhere to (Figure 15C). According to the authors, this could be a possible reason for the observed 2D flat morphology of pMSCs in the culture interval 10–20 min. At longer culture times, reassembly of cytoskeletal filaments caused a change of 2D to 3D cell morphology. However, due to the promoted hydrophilicity and enhanced number of oxygen-containing groups on the plasma-treated scaffolds, the cells still stretched considerably on the nanofibrous surface in comparison to the pristine sample (Figure 15D).

**Figure 16:**
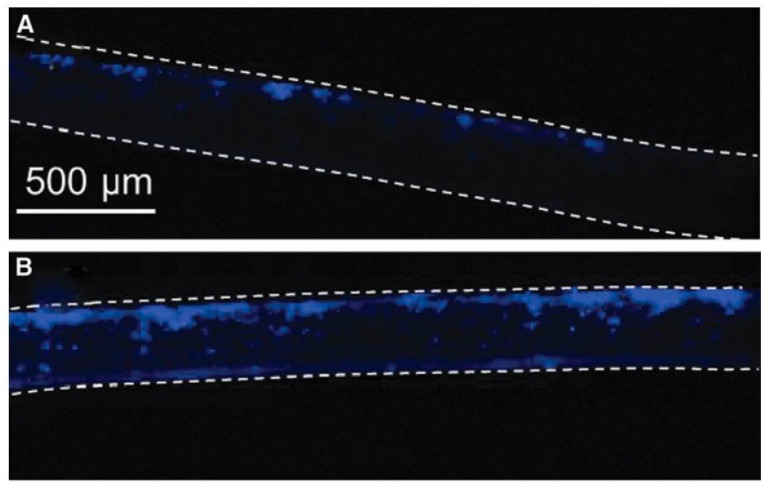
Cross-sectional images of (**A**) untreated and (**B**) Ar plasma-modified scaffolds sustained after in vitro study with BAECs in the serum medium for five days. Top and bottom scaffold surfaces are marked by dashed lines—reproduced with permission from [248]. Copyright Liebert, 2013.

Similar to PCL, also the impact of an Ar plasma treatment has been investigated on PLLA [247]. Surface treatment of PLLA microfibrous meshes was performed using a vacuum RF plasma reactor (type CCP). Ar plasma modification was conducted for 2 min under conditions of 30 W power. In this case, the WCA value was found to decrease from 116° to 85° upon plasma treatment without physically changing the PLLA fiber morphology. In this particular paper, however, no enhancement in surface oxygen content was detected on the plasma-treated PLLA scaffold, which is in contrast to what is typically described in the literature. Cell studies revealed that both bovine aorta endothelial cells (BAECs) and bovine smooth muscle cells (BSMCs) adhered more and spread better on the plasma-treated scaffold compared to the untreated sample. Additionally, the applied Ar plasma treatment also strongly increased the proliferation rate of both cell types compared to the control scaffold. The authors have also conducted in vitro and in vivo studies to investigate the impact of the Ar plasma modification on the infiltration of cells inside the 3D PLLA scaffolds [247]. Figure 16 contains representative cross-sectional images of PLLA scaffolds cultured with BAECs, which have been stained with 4′-6-diamidino-2phenylindole (DAPI) after 5 days of incubation. The cells were cultured at the scaffold’s top surfaces. In case of the untreated scaffold surface, the density of the cells was low and cell infiltration in the scaffold was very limited (Figure 16A). However, after Ar plasma modification considerably more cells were present at the top surface of the scaffold and cell infiltration in the scaffold was higher (Figure 16B). As such, besides increasing cell spreading and cell growth, the Ar plasma treatment also promoted in vitro cell infiltration in the scaffold. It is most likely that the scaffold was homogenously modified by the Ar plasma treatment due the high scaffold porosity, which can, in turn, explain the increased cell infiltration in the Ar plasma-treated scaffold. The authors also conducted in vivo experiments by implanting the scaffolds under the skin of Sprague-Dawley rats. The obtained results revealed that the Ar plasma treatment was also able to improve the PLLA scaffold bioactivity in vivo resulting in enhanced cell infiltration. However, additional scaffold modification with bioactive molecules will still be required to improve angiogenesis, tissue-specific responses and regeneration [247].

Besides PCL and PLLA, also electrospun silk fibroin scaffolds have been exposed to a low-pressure RF discharge generated in O_2_ [249]. Similar as for PCL and PLA scaffolds, the authors observed a strong increase in surface hydrophilicity upon O_2_ plasma treatment: the WCA value of the silk fibroin scaffolds decreased from 115° to 39° after a 10 s plasma treatment, while a 30 s plasma exposure resulted in a decrease in WCA to 0° due to rapid penetration of the water drop into the nanofibrous structure. This increased wettability was also in the case of silk fibroin fully ascribed to polar oxygen-containing group incorporation at the scaffold surface, as the nitrogen content remained unaffected [249]. The authors also observed that the nanofiber morphology was not affected by the performed plasma modification. However, no plasma exposure time was mentioned for the SEM image used to prove this statement. Consequently, it is impossible to know at which plasma exposure times exactly the plasma treatment is not destructive. As the authors envisioned skin TE applications for their scaffolds, they also examined the behavior of normal human epidermal keratinocytes (NHEK) and normal human epidermal fibroblasts (NHEF) on pristine and O_2_ plasma-treated silk fibroin scaffolds. For this purpose, a plasma treatment time of 3 min was selected, although it is not clear whether this prolonged treatment time is not resulting in nanofiber damage. The conducted cell assays revealed that the initial number of adhered NHEF and NHEK cells was lower on the O_2_ plasma-exposed sample compared to the untreated silk fibroin scaffold. In contrast, three days and seven days post-cell seeding, the numbers of NHEF and NHEK cells proliferating on the plasma-treated scaffolds were significantly higher compared to the untreated sample. The authors suggested that this higher cellular activity was caused by increased scaffold surface hydrophilicity.

**Figure 17:**
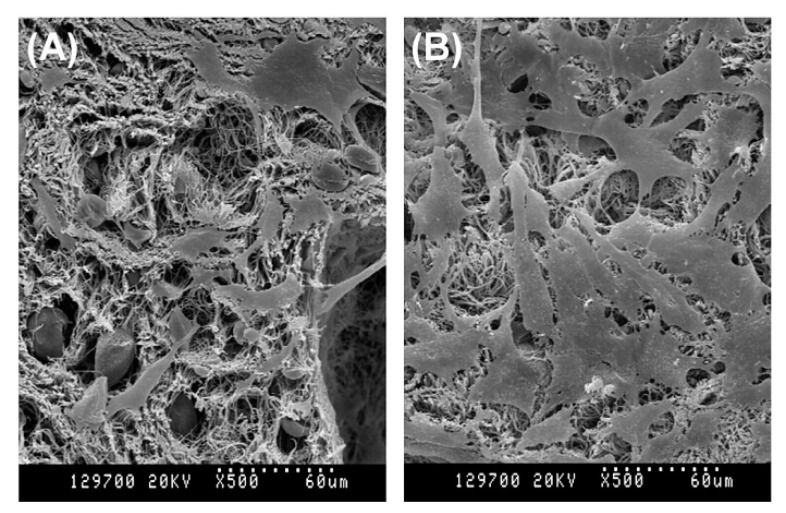
SEM micrographs of nHAC-kn cultured for seven days onto untreated (**A**) and Ar plasma-modified (**B**) 3D porous nanofibrous silk fibroin scaffolds—reproduced with permission from [249]. Copyright Elsevier, 2008.

Instead of an O_2_ plasma, Baek et al. have employed a microwave (MW)-induced plasma sustained in Ar at atmospheric pressure to modify the surface properties of a nanofibrous silk fibroin 3D scaffold, obtained by electrospinning followed by salt leaching [249]. The plasma exposure time was fixed at approximately 12 s in this specific work. Although limited attention was paid to the scaffold surface characterization, an extended examination of the cellular behavior of neonatal human knee articular chondrocytes (nHAC-kn) was performed. The authors observed that the attachment and proliferation of the chondrocytes was significantly increased on the plasma-modified scaffolds, except for a one-day post-cell seeding. Additionally, they also examined the morphology of the nHACs growing on the scaffolds seven days post-seeding using SEM. The cells on the pristine scaffold spread only locally on the surface, as shown in Figure 17A. In contrast, the cells on the plasma-treated scaffolds almost fully covered the scaffold surface, while maintaining the natural original morphology of articular chondrocytes (Figure 17B). Based on these obtained results, the authors suggested that the plasma-modified scaffolds can be potentially used as cartilage TE scaffolds.

Instead of using pure oxygen for surface activation, air plasma, which is less expensive, can be just as effective. Two separate studies conducted by De Valence et al. [250] and Prabhakaran et al. [251] focused on a vacuum RF plasma sustained in air and operating at 30 W power for the surface activation of PCL scaffolds. Both papers reported a decrease in WCA from 122° to 0° upon a 1 min air plasma treatment. Although air is mainly consisting of nitrogen, the performed plasma treatments were found to result in a negligible incorporation of nitrogen on the surface of electrospun PCL scaffolds. However, a significant incorporation of oxygen was observed (increase of 4–12%), which is considered to be the main responsible for the highly increased surface wettability. Unfortunately, the air plasma treatment was also found to have a negative effect on the tensile strength of the PCL scaffolds, while the elongation at break remained unaffected [251]. De Valence et al. also examined the growth of Rhomboid smooth muscle cells on control and air plasma-treated PCL scaffolds, but observed no statistically significant improvements in cell adhesion and proliferation upon plasma treatment. Nevertheless, the cells were found to have a more spread-out morphology in comparison to the untreated electrospun PCL scaffold [250]. In contrast, Prabhakaran et al. [251] showed a significantly improved proliferation of Schwann cells by conducting an air plasma treatment on electrospun PCL scaffolds. These results thus show that cell-surface interactions are not only depending on the applied plasma treatment, but also on the cell type which is being investigated.

**Figure 18:**
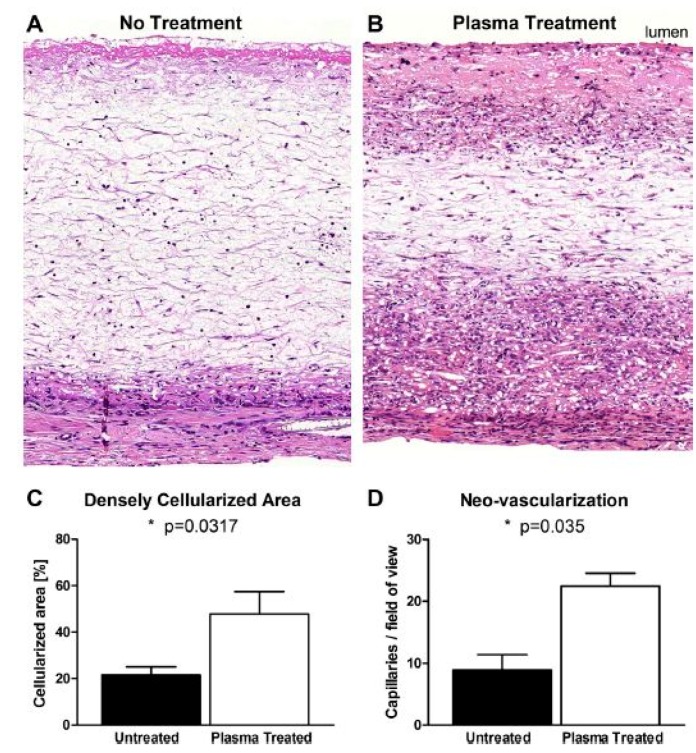
Visualization of the effect of air plasma treatment on tubular PCL scaffolds after vascular implantation: plasma modification not only promoted the invasion of the cells in the scaffold wall (**A**,**B**), but also enhanced the densely cellularized area (**C**) and the number of capillaries in the scaffold wall (**D**)—reproduced with permission from [250]. Copyright Elsevier, 2013.

Similar to what was done by Cheng et al. on PLLA scaffolds [247], De Valence et al. also compared the in vivo performance of pristine and air plasma-modified tubular PCL scaffolds [250]. In a first step, both scaffold types were subcutaneously implanted into Sprague-Dawley rats and after three weeks, the implants and the surrounding tissues were removed from the rats. In the next step, the implants were fixated and embedded in paraffin, after which the middle of the implant was cut into sections in the transversal direction (thickness of 4 µm). These 4 µm thick sections were subsequently stained with hematoxylin-eosin (H&E) and scanned at a high resolution. After three weeks of subcutaneous implantation, it was observed that the lumens of both untreated and plasma-treated implants towards the center of the tubes were still void of tissue, resulting in cell infiltration only from the outer side. At the tissue-scaffold interface in the case of both scaffold types, a thin and continuous layer of large cells was observed. Moreover, for both implants, no large inflammatory responses and no thick fibrin capsules around the scaffolds were observed. Moreover, a thick layer of cells, including fibroblasts, macrophages, some neo-capillaries and scarce lymphocytes infiltrated both scaffolds from the outside. However, this infiltrated cell layer presented a considerably larger penetration depth, makes out a larger area of the total scaffold wall and has a significantly higher cell density in case of the plasma-treated scaffold compared to the untreated sample, both in terms of depth of penetration depth and of area fraction of the total scaffold wall. Besides subcutaneous implantation, the pristine and plasma-modified tubular PCL scaffolds were also implanted in the abdominal aorta of rats to act as vascular TE scaffolds. After three weeks of implantation, the vascular implants were extracted, fixed, cut in the longitudinal direction, fixed in paraffin and cut into 4 µm thick sections which were also stained with H&E and scanned at high resolution. In Figure 18, the untreated and air plasma-modified vascular scaffolds after H&E staining are depicted together with the quantitative results of the cellularized area and the capillaries per field of view. For both scaffolds under study, cell movement from the adventitia toward the scaffold wall assisted in the formation of a neo-tissue, but this formation was more pronounced in the case of the plasma-modified scaffold (Figure 18A,B). In addition, a similar cellular infiltrate as observed on the subcutaneous implants, was also seen. At the adventitial edges of the scaffolds, a continuous lining of large cells was observed for both scaffold types and a dense infiltrate of fibroblasts, macrophages, neo-capillaries and some lymphocytes invaded the scaffolds from the adventitia. Quantification of this infiltrate (Figure 18C) showed that the average cell penetration depth was considerably enhanced by the performed plasma modification process. The authors also performed quantification of the neo-vascularization (Figure 18D) and observed a significant increase in capillaries per field of view in the case of the plasma-treated PCL tubular scaffold. In addition to the dense cell infiltrate, isolated fibroblasts and lymphocytes could also be found throughout the thickness of both scaffold types, while no transanastomotic migration of smooth muscle cells into the synthetic scaffolds was observed. It was therefore concluded that the conducted air plasma treatment prompted the formation of the neo-tissue and accelerated the tissue regeneration process. Plasma modification was therefore considered by the authors as a simple and effective method to improve the performance of TE scaffolds in vascular applications.

Besides O_2_, Ar and air plasmas, which are known to result in the introduction of oxygen functional groups, also nitrogen-containing plasmas (N_2_, NH_3_, He/NH_3_…) can be employed for the surface activation of nanofibrous TE scaffolds. Different authors have already focused on examining the impact of a nitrogen (N_2_) plasma on the surface characteristics and cellular interactions of electrospun scaffolds [180,252,253,254,255]. In 2014, Dolci et al. [252] employed a linear corona discharge sustained in N_2_ at atmospheric pressure to modify the surface properties of electrospun PLLA scaffolds [256]. Plasma treatments were performed in the open air and plasma exposure time was fixed at 20 s. The performed plasma treatment did not induce modifications to the morphology, the thermal characteristics and the mechanical properties of the PLLA scaffolds. The only appreciable effect of the N_2_ plasma treatment was a slight decrease in the elastic modulus associated with a small increase of deformation at break, attributed to a slight loss of rigidity. Similar to O_2_ plasmas, the N_2_ plasma treatment drastically lowered the WCA value of the PLLA scaffolds from 120° for the pristine sample to values close to 0° (as the water drop instantaneously penetrated into the scaffold) for the plasma-treated mesh. According to the authors, the enhanced PLLA surface hydrophilicity is due to the introduction of oxygen-containing functionalities on the scaffold surface, although XPS measurements are not included to prove this statement. The authors did, however, detect a higher content of oxygen-containing groups (namely COOH groups) on N_2_ plasma-treated PLLA scaffolds through chemical conjugation reactions followed by functionalization with the fluorescent fluorescein isothiocyanate molecule. This peculiar oxygen incorporation can be explained by the fact that the N_2_ plasma treatment in this work is conducted in open air. Consequently, it is reasonable to assume that air entrainment in the plasma region causes the formation of highly reactive oxygen species responsible for the introduction of oxygen-containing functionalities at the material surface. In the final stage of their research, Dolci et al. also compared the behavior of mouse embryonic fibroblasts (MEFs) on untreated and N_2_ plasma-modified PLLA scaffolds [256]. MEFs cultured on the plasma-modified scaffold showed a more elongated and dendritic morphology and a higher vitality than MEFs cultured on the untreated scaffold.

More recently, Asadian et al. have examined the surface activation of PCL and chitosan (CS)/polyethylene oxide (PEO) scaffolds using a medium pressure DBD sustained in nitrogen [180,253]. In contrast to the work of Dolci et al. [256], N_2_ plasma treatments of the scaffolds were thus performed in a closed chamber. For both scaffold types, the discharge power was fixed at 4.6 W and the plasma exposure time was varied between 5 and 60/70 s for the CS/PEO and PCL scaffolds respectively. In case of PCL, the WCA value was found to decrease from 135° to 25°, while for the more hydrophilic CS/PEO scaffolds, the WCA decreased from 57° to approximately 15°. The applied N_2_ plasma treatments also caused no morphological changes to the PCL and CS/PEO scaffolds. XPS analysis revealed that besides the O/C ratio, also the N/C ratio was significantly increased on the plasma-modified CS/PEO scaffolds, showing that both oxygen- and nitrogen-containing functionalities were introduced on the CS/PEO scaffolds’ surfaces. In contrast, in case of PCL scaffolds, only the introduction of nitrogen-containing functionalities was observed. XPS derivatization reactions also revealed that the N_2_ plasma treatments only resulted in a low incorporation of primary amine (NH_2_) groups. The authors also examined the behavior of HFFs on pristine and N_2_ plasma-modified PCL and CS/PEO scaffolds and observed significantly better HFF attachment and proliferation on the plasma-modified samples.

As different studies have observed superior cell growth on NH_2_-enriched surfaces in comparison to other functionalized surfaces [255,257], efforts have also been undertaken to specifically graft NH_2_ functional groups using NTPs on nanofibrous TE scaffolds. For this purpose, plasmas sustained in ammonia (NH_3_) have been commonly applied, although also in this case only a small percentage of nitrogen is generally incorporated as NH_2_ groups. In a recent study, Ivanova et al. [244] have exposed PCL scaffolds to a vacuum RF discharge sustained in NH_3_ using a discharge power of 50 W (plasma exposure time: 120 s). In this case, the WCA was found to only decrease with 20°, which was significantly lower compared to the ones observed when they applied Ar and O_2_ plasmas to the same PCL scaffolds (50° and 90° respectively). XPS analyses also revealed that the conducted NH_3_ plasma modification resulted in a slight reduction in oxygen content combined with only a low amount of nitrogen incorporation. Unfortunately, no specific information was provided on the amount of NH_2_ groups that were incorporated on the PCL nanofibrous surface. The low introduction of polar functionalities can, however, explain the rather poor increase in surface hydrophilicity of the PCL scaffolds upon NH_3_ plasma treatment. The authors also concluded that an O_2_ plasma seems to be the most efficient in increasing the PCL surface hydrophilicity as this plasma results in the highest incorporation of oxygen functional groups.

**Figure 19:**
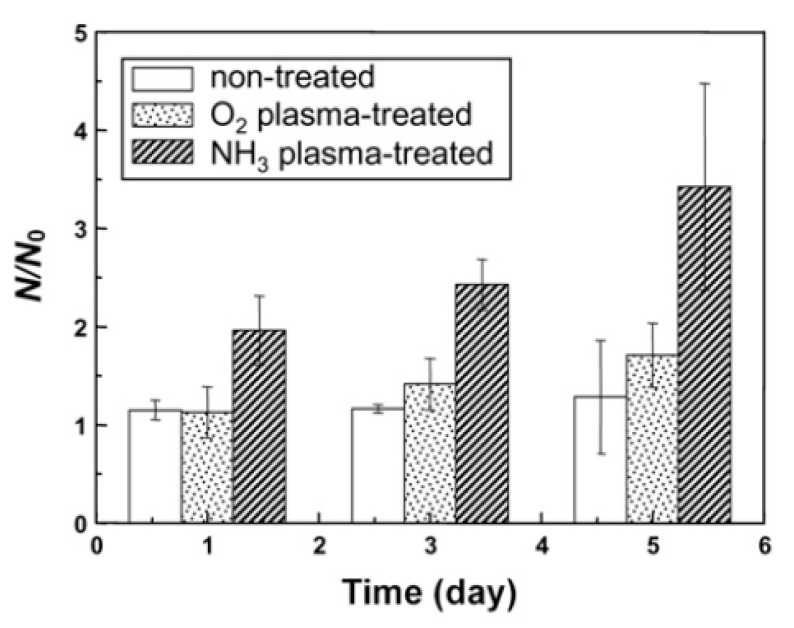
Growth of 3T3 fibroblasts after seeding on various PLGA nanofibers after one, three and five days (plasma exposure time not specified in the paper)—reproduced with permission from [260]. Copyright Springer, 2007.

NH_3_ plasma treatments have also been applied to PLGA nanofibrous scaffolds by Park et al. [258,259]. In 2007, Park et al. examined the effects of a low-pressure NH_3_ discharge on PLGA nanofibers and also used the same plasma set-up sustained in O_2_ to modify the surface properties of the PLGA nanofibers. Plasma exposure times were varied between 30 and 300 s. Unfortunately, no information on applied discharge powers could be found in the publication. When exposed to the NH_3_ and O_2_ plasmas for 30 s, no significant changes in the morphology of the PLGA nanofibers were observed. With increasing plasma exposure times up to 300 s, the dimensions and morphology of the PLG nanofibers remained unaffected when applying the NH_3_ plasma treatment. In contrast, PLGA nanofibers exposed to O_2_ plasma for 300 s slightly lost their nanofibrous structure. This observation could suggest that an O_2_ plasma is more destructive than an NH_3_ plasma, however, as no information is given on the applied discharge power, the observed differences in damage may also be attributed to the use of a higher discharge power in case of O_2_ plasma. To investigate changes in surface hydrophilicity, the WCA evolution as a function of plasma exposure time was also obtained using both discharge gases (NH_3_ and O_2_). In contrast to the work of Ivanova et al. [244], the ammonia plasma most effectively enhanced the surface hydrophilicity of the PLGA nanofibers: the WCA of the PLGA nanofibers decreased from 134° to 115° and 45° after a 300 s treatment with O_2_ plasma and NH_3_ plasma respectively. XPS analysis also found that the O_2_ plasma treatment resulted in the incorporation of oxygen functional groups, while in the case of NH_3_ plasma treatment, up to 3 at % of nitrogen was grafted to the PLGA nanofibers. Unfortunately, information on the amount of incorporated NH_2_ groups was again not given. The authors also examined the adhesion and proliferation of 3T3 fibroblasts on the O_2_ and NH_3_ plasma-treated PLGA samples using an MTS assay and the results they obtained are shown in Figure 19. Cellular adhesion to PLGA was significantly improved by plasma treatment, especially when using the NH_3_ plasma treatment. Additionally, cells seeded on NH_3_ plasma-treated nanofibers proliferated significantly more than those on untreated or O_2_ plasma-treated nanofibers, as can be clearly seen in Figure 19. This enhanced cell growth upon NH_3_ plasma modification may be attributed to the more hydrophilic surface properties of the NH_3_ plasma-treated PLGA nanofibers.

In an additional paper, Park et al. more closely examined the influence of plasma exposure time on the cellular behavior of 3T3 fibroblasts on NH_3_ plasma-treated PLGA scaffolds using the same plasma reactor as mentioned above [259]. However, in this case, the applied discharge power was mentioned to be 100 W and the plasma exposure time was set to 30 s, 60 s, and 180 s. Interestingly, the authors observed that prolonged plasma treatment times resulted in reduced cell viability, however, a clear explanation for this peculiar behavior was not given [259].

Finally, Cheng et al. also exposed PLLA microfibrous scaffolds to a vacuum RF plasma reactor (type CCP) using an optimized NH_3_ plasma treatment [247]. In a first step, an NH_3_ plasma treatment was conducted for 5 min at 50 W using a mixture of Ar and NH_3_, after which a 30 s H_2_ plasma treatment at 10 W was performed to maximize the amount of incorporated NH_2_ groups at the scaffold surface. Hereafter, this modification procedure will be attributed to Ar-NH_3_/H_2_ treatment. These authors also conducted a pure Ar plasma treatment on the same microfibrous PLLA scaffolds, as extensively described previously. Upon Ar-NH_3_/H_2_ plasma treatment, the WCA value of the PLLA scaffolds was found to be considerably lower than in the case of Ar plasma treatment as WCA values close to 0° were observed. XPS analysis revealed the incorporation of nitrogen groups on the Ar-NH_3_/H_2_ plasma-treated scaffolds and the amount of incorporated NH_2_ groups were also quantified (NH_2_/C ratio equal to approximately 1.5%). Cell studies revealed that BAECs and BSMCs adhered and elongated more on the Ar-NH_3_/H_2_ plasma-treated scaffold than on the Ar plasma-treated sample. In contrast, the applied Ar plasma modification was more efficient in improving the growth rate of both BAECs and BSMCs than the Ar-NH_3_/H_2_ plasma modification. A possible reason for the various proliferation rates after these plasma treatments is that the surface chemical functionalities introduced by the Ar plasma not only improved wettability but also caused serum protein adsorption in a way which is more desirable for cell growth, despite the fact that the Ar-NH_3_/H_2_ plasma treatment resulted in a higher initial cell spreading. It seemed that Ar surface activation was more favorable to enhance cell proliferation than surface functionalization with −NH_2_ groups, which is widely known to improve surface biocompatibility. Obviously, more studies are needed to completely understand the influence of these plasma conditions on the biochemical characteristics of PLLA scaffolds.

A summary of all conducted in vitro cell studies on plasma-activated nanofibrous scaffolds can be found in Table 7, arranged according to the envisioned TE application.

### 4.3. Protein Immobilization or Adsorption on Plasma-Activated Nanofibrous Scaffolds

It has already been observed that preliminary protein adsorption upon initial exposure of nanofibers to media culture (called protein corona) highly depends on the nanofiber surface chemical cues. This protein corona behavior is highly able to control the cell fate by altering/initiating intra- and inter-cellular signaling pathways [180,254]. In an effort to more precisely control cellular interactions, a large number of proteins have already been covalently immobilized on plasma-activated nanofibrous scaffolds using carbodiimide coupling of a carboxylic (COOH) group with an NH_2_ group, [242,250,271]. Depending on the immobilization conditions, this strategy typically has a minimal effect on the conformation of the protein structure, meaning that it is possible to retain the functional efficiency of the immobilized proteins. Through tailored protein immobilization, more specific desired cellular responses can be achieved [272,273] compared to just plasma-activated nanofibrous scaffolds, making this a very popular approach to functionalize TE scaffolds. In contrast to covalent immobilization, a dip coating procedure can also be used in which proteins are physically adsorbed on nanofibrous scaffolds [274,275].

Among different proteins, which can be immobilized or adsorbed on plasma-activated nanofibrous scaffolds, collagen is by far the most widely examined [135,250,276,277,278]. In an effort to develop an ideal bone graft substitute, Shabani et al. grafted collagen I onto the surface of a plasma-activated polyethersulpone (PES) nanofibrous scaffold to promote the scaffold osteoconductivity [272], since the pristine polymer shows a low cell affinity for cell infiltration. In this particular work, plasma modification of the PES scaffold was carried out in pure O_2_ using a low-pressure discharge for 5 min. Subsequently, collagen I was covalently bond to the plasma-activated surface using a 2 step process: first, the plasma-treated samples were immersed in a 1-ethyl-3-(3-dimethylaminopropyl)carbodiimide/N-hydroxysuccinimide (EDC/NHS) solution (5 mg/mL) for 12 h, after which they were subsequently immersed in a 1 mg/mL collagen I solution overnight. WCA analysis showed that the O_2_ plasma treatment resulted in a decrease in WCA value from 132° to 0°, while after collagen grafting the WCA value remained at 0°. Morphology results also revealed that the average fiber diameter and porosity of the PES scaffolds were not affected by the O_2_ plasma treatment. In contrast, after collagen grafting, which was confirmed using Fourier-transform infrared spectroscopy (FTIR), a small decrease in porosity and an increase in average fiber diameter could be observed. The authors also compared the behavior of unrestricted somatic stem cells (USSCs) on the untreated, plasma-modified and collagen-grafted PES scaffolds and observed the following: (1) the USSCs proliferated significantly better on the collagen-grafted scaffolds, (2) collagen-grafted scaffolds had the highest capacity to support osteogenic differentiation of the USSCs, which was confirmed via the assessment of osteogenic markers and (3) USSCs infiltrated best into the collagen-grafted scaffolds as evidenced from histologic examination. Consequently, the authors concluded that collagen-grafted PES scaffolds can be suitable for potential 3D bone grafts with high capacity for bone healing and regeneration in vivo. Additionally, also mouse embryonic stem cells (mESCs) cultured on the collagen-coated PES scaffolds were found to have a typical undifferentiated morphology, enhanced proliferation, stable diploid normal karyotype and continued expression of stemness and pluripotency-associated markers in comparison to the untreated PES scaffolds [279]. In another paper focusing on the covalent grafting of collagen on O_2_ plasma-treated PES scaffolds written by Shabani et al. [278], a slightly different experimental procedure was used. In this particular paper, a plasma treatment of 10 min instead of 5 min was applied as well as an immersion time of 6 h instead of 12 h in the EDC/NHS solution. Also in this paper, it was observed that USSCs showed very high infiltration into the collagen-grafted scaffolds, as qualitatively observed from SEM imaging. Exactly the same plasma-based strategy as the one first mentioned in this paragraph has been also applied by Seyedjafari et al. to covalently graft collagen I on electrospun PLLA scaffolds [280]. In this paper, the behavior of USSCs was also examined on untreated, plasma-modified and collagen-grafted PLLA scaffolds and similar conclusions as mentioned for the PES scaffolds were drawn. Islami et al. have also examined the covalent coupling of collagen I on plasma-activated PLLA scaffolds using carbodiimide chemistry showing excellent attachment, outgrowth and proliferation of CD 133+ cells on the collagen-coated PLLA scaffolds [281].

**Figure 20:**
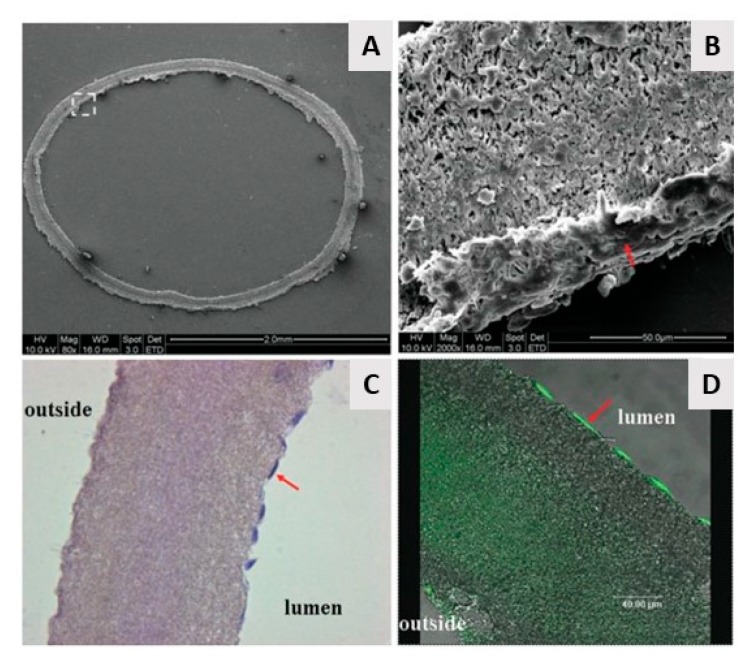
Visualization of the proliferation of HCAECs on plasma-modified tubular nanofibrous P(LLA-CL) conduits 10 days after cell culturing: (**A**,**B**) SEM micrographs of the cell-seeded scaffolds: (**A**) cross-section of the scaffold, (**B**) higher magnification image of the quadrangle shown in (**A**); (**C**) cross-section of the scaffold after H&E staining and (**D**) cross-section of the scaffold after immunostaining of PECAM-1. Original magnification: (**A**) 80×, (**B**) 2000×, (**C**) 200× and (**D**) 630×. Scale bar: (**A**) 500 µm, (**B**,**C**) 2 mm and (**D**) 100 µm—reproduced with permission from [135]. Copyright Wiley, 2009.

Instead of using carbodiimide chemistry as was done in the papers described above to covalently bind collagen to a nanofibrous surface, collagen can also be physically adsorbed on plasma-activated surfaces. In 2010, Feng et al. carried out NH_3_ plasma treatments using a vacuum RF discharge (type ICP) on random and aligned PLLA nanofibers after which the scaffolds were submerged into a collagen I solution (300 μg/mL) in 0.1 M HCl at 4 °C for 12 h [242]. At a pH value of 7.2, the NH_2_ groups present on the NH_3_ plasma-treated nanofibers displayed positive charges while the carboxyl groups of collagen I displayed negative charges. Consequently, electrostatic interactions between these opposite charges allow stable adsorption of collagen onto the aligned PLLA nanofibers. Blood outgrowth endothelial cells (BOECs) cultured on the collagen-coated random and aligned PLLA nanofibers showed excellent cell attachment and viability and maintained their endothelial phenotype. A similar strategy as the one used by Feng et al. [242] was also followed by He et al. to physically adsorb collagen I on air plasma-treated flat and tubular scaffolds fabricated from a 70:30 PLLA/PCL co-polymer [282,283]. The air plasma treatment was conducted using a vacuum RF discharge (type ICP) at 30 W for 5 min for flat random and aligned scaffolds and 15 min for the tubular scaffolds. These authors also observed that the spreading, viability and attachment of human coronary artery endothelial cells (HCAECs) was superior on the collagen-coated P(LLA-CL) flat scaffolds in comparison to the untreated scaffolds. Moreover, it was also observed that the phenotype of the endothelial cells was also preserved on these collagen-coated flat samples [283,284]. In case of the tubular nanofibrous P(LLA-CL) scaffolds, HCAECs were also found to nicely elongate on the lumen of the scaffolds and these cells were observed to achieve sub-confluency in only 1 day after cell culturing on the inside of the tubes. Additionally, after a 10 day culture period, the phenotype of the HCAECs was still preserved and a thin layer of HCAECs was covering the lumen of the conduits, as depicted in the images shown in Figure 20 [135]. From all the above-mentioned results, it can thus be concluded that collagen-coated PLLA and P(LLA-CL) nanofibers can be very suitable scaffolds for vascular TE applications.

Collagen I has also been physically adsorbed on the surface of nanofibrous poly (hydroxybutyrate-co-hydroxyvalerate) (PHBV) scaffolds by Ai et al. [285] and Rezaei-Tavirani et al. [284] to improve its cell affinity as this polymer is a hydrophobic polyester possessing poor cellular interactions. After applying an MW plasma sustained in O_2_ for 30 or 60 s at a power of 100 W, the plasma-activated scaffolds were submerged in a collagen I solution (15 mg/mL in 50 mM acetic acid) for 2 h at 50 °C. WCA analysis showed that the performed plasma treatments resulted in an increased PHBV surface wettability as the WCA was found to decrease from 67° to approximately 56–58° after plasma treatment. Cellular investigations using USSCs also revealed better cell adhesion, growth and cell viability on the collagen-coated PHBV nanofibers compared to their untreated counterparts, showing that even minor changes in wettability can have a profound impact on cell-surface interactions.

**Figure 21:**
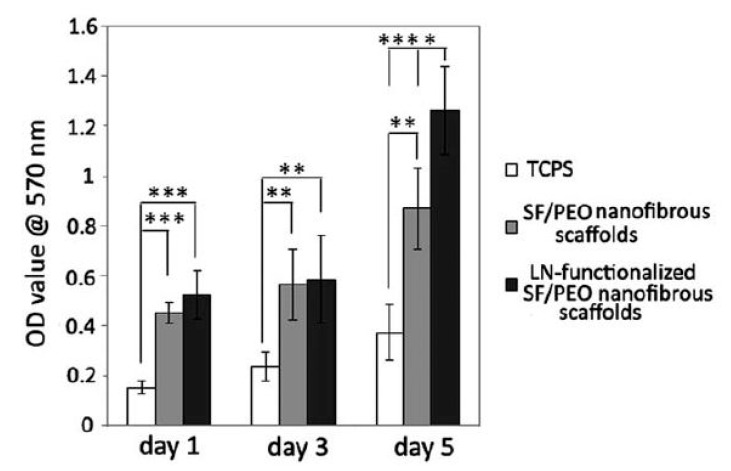
Proliferation of Schwann cells seeded on TCPS, electrospun silk fibroin (SF)/ polyethylene oxide (PEO) nanofibrous scaffolds and electrospun laminin (LN)-functionalized SF/PEO nanofibrous scaffolds at day 1, 3 and 5 (*p* < 0.01 (**), *p* < 0.005 (***) and *p* < 0.0001 (****)—reproduced with permission from [270]. Copyright Wiley, 2018.

After collagen grafting, laminin immobilization is the second most investigated protein that has been grafted on plasma-activated nanofibrous scaffolds. In 2008, Koh et al. have examined in detail the grafting of laminin on nanofibrous PLLA scaffolds after performing a vacuum RF plasma treatment in air (30 W, 5 min) [285]. Two approaches were used to graft laminin on the plasma-treated PLLA scaffolds: (1) covalent immobilization of laminin using carbodiimide chemistry and (2) physical adsorption of laminin by immersing the plasma-activated samples into a laminin solution. The authors observed a homogeneous distribution of laminin on the PLLA nanofibers for both applied grafting strategies and showed that for each approach, laminin was added to the surface of the PLLA scaffold as indicated by the presence of a nitrogen peak in the XPS survey spectra. The behavior of nerve PC12 cells on the differently prepared PLLA scaffolds was also examined by the authors and it was observed that the laminin-PLLA nanofibers showed better cell viability compared to the unmodified PLLA sample. Additionally, the laminin-modified scaffolds also supported the attachment and proliferation of PC12 cells and can thus serve as effective substrates to enhance nerve regeneration. Moreover, it has been found that silk fibroin (SF) shows great potential for TE applications, however, this material has a limited cell affinity due to the lack of cell recognition sites. As such, laminin has also been physically adsorbed on O_2_ plasma-treated SF/PEO scaffolds envisioning nerve TE applications by Rajabi et al. [272]. The absorbance level of dissolved formazan (MTS assay) was measured as an indication of the proliferation of Schwann cells seeded on tissue culture polystyrene (TCPS), untreated SF/PEO nanofibrous scaffolds and laminin-functionalized SF/PEO nanofibrous scaffolds and the results are shown in Figure 21. As it is shown, cell viability and proliferation were not statistically different on day 1 and day 3 for all investigated sample conditions. Only after five days of incubation, the number of cells was found to be significantly increased compared to the earlier examined days. Figure 21 also reveals that the Schwann cells proliferated better on the untreated SF/PEO scaffolds compared to TCPS, while the best cell proliferation was observed on the laminin-functionalized scaffolds, especially after a five-day culture. Consequently, Rajabi et al. [272] came to the same conclusion as Koh et al. [285], namely that laminin-coated scaffolds can be excellent candidates for nerve TE applications.

In a more recent study by Sahebalzamani et al., laminin has been physically adsorbed on CO_2_ plasma-treated electrospun PCL scaffolds [286]. The conducted plasma treatment led to a reduction of the WCA value from 112° to 67° due to the introduction of oxygen-containing functionalities. The subsequent dip coating in a laminin solution resulted in a further decrease in WCA value down to 30°. Similar as the previously mentioned studies, the adsorbed laminin was found to be responsible for significantly improved fibroblast viability compared to the plasma-treated electrospun PCL scaffold [286].

**Figure 22:**
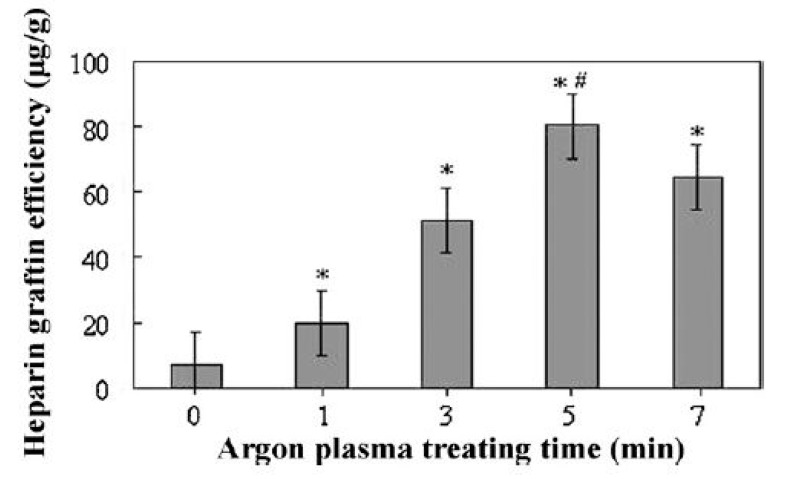
Grafting performance of heparin to silk fibroin scaffolds as a function of Ar plasma treatment time (* *p* < 0.05, significant different between the grafting efficiency of plasma-treated scaffolds and untreated scaffolds; # *p* < 0.05, significant different between the performance at an Ar plasma exposure time of 5 min compared to an Ar plasma exposure time of 1, 3 and 7 min)—reproduced with permission from [273]. Copyright Elsevier, 2011.

Besides laminin, also heparin has been physically adsorbed on the surface of plasma-activated nanofibrous scaffolds [271]. In a study written by Wang et al., electrospun silk fibroin scaffolds were first exposed to a low-pressure Ar plasma, after which the anti-coagulant heparin was grafted to the plasma-activated scaffolds by immersing the samples in a 5 mg/mL heparin sodium salt aqueous solution for 30 min [271]. This strategy was followed in an effort to improve the anti-coagulative properties and biocompatibility of the scaffolds. Results clearly showed that without the use of a plasma pre-treatment, only a few heparin particles were seen on the scaffolds. On the contrary, on the Ar plasma-treated silk fibroin scaffolds (90 W, 5 min), a significant mass of heparin particles, which were homogeneously distributed along the scaffold, was present. Moreover, even after washing the scaffolds in an ultrasonic water bath, most of the heparin particles remained on the plasma-treated scaffold surface indicating strong interactions between the scaffold surface functional groups and the heparin granules. The successful heparin grafting on the plasma-treated scaffolds was also further confirmed using FTIR and XPS analysis. As the plasma treatment time can also significantly affect the grafting efficiency, the authors also examined the heparin grafting efficiency as a function of Ar plasma treatment time and the obtained results are shown in Figure 22. This image clearly shows that the heparin grafting efficiency gradually increases with increasing plasma exposure time until a maximum value was observed at a plasma exposure time of 5 min. In contrast, a longer treatment time of 7 min resulted in less efficient heparin grafting.

In the second stage of their research, Wang et al. examined the in vitro antithrombogenicity of the untreated and heparin-grafted silk fibroin scaffolds (Ar plasma: 90 W, 5 min) and observed significantly enhanced anti-coagulative properties as a result of the heparin modification. Additionally, L929 fibroblasts and vascular endothelial cells (VECs) were utilized to assess in vitro cell adhesion and proliferation on the scaffolds before and after heparin grafting. It was observed that cells adhered and proliferation much superior on the heparin-grafted scaffolds compared to the pristine samples. Finally, the authors also examined the in vivo performance of their developed silk fibroin scaffolds by implanting the scaffolds on the back of rats. It was observed that the scaffolds were covered by regenerated tissue. In addition, the heparin grafted scaffold (Ar plasma: 90 W, 5 min) showed considerably less inflammation and no rejection by the host as macrophages, neutrophils, and lymphocytes were not seen. Electrospun heparin-grafted silk fibroin nanofibrous meshes are thus excellent candidates for TE applications, including vascular grafts, wound dressings and skeletal muscle substitutes.

Besides the above-mentioned proteins, a few research papers have also been focusing on the grafting of elastin [287], gelatin [288,289], fibronectin [281] and soluble eggshell membrane protein [290] on nanofibrous scaffolds. Also, in these cases, better cellular interactions were observed on the protein-grafted scaffolds, consequently, the grafting of these less examined proteins will not be described in detail in this review paper.

### 4.4. Grafting of Inorganic Particles on Plasma-Activated Nanofibrous Scaffolds

Incorporating inorganic particles in nanofibrous scaffolds and deposition of a thin inorganic layer on a scaffold surface to obtain excellent performance in bone tissue regeneration are highly promising ways for biomineralization of the scaffold bulk or scaffold surface respectively. Different inorganic molecules have already been utilized for this purpose, including hydroxyapatite (HA), CaCO_3_ and TiO_2_ [291,292,293,294]. The deposition of the previously mentioned species on as-fabricated electrospun scaffolds has already been widely examined. However, very recently, research is also been conducted on the deposition of these species on plasma-activated nanofibrous scaffolds [244,295].

**Figure 23:**
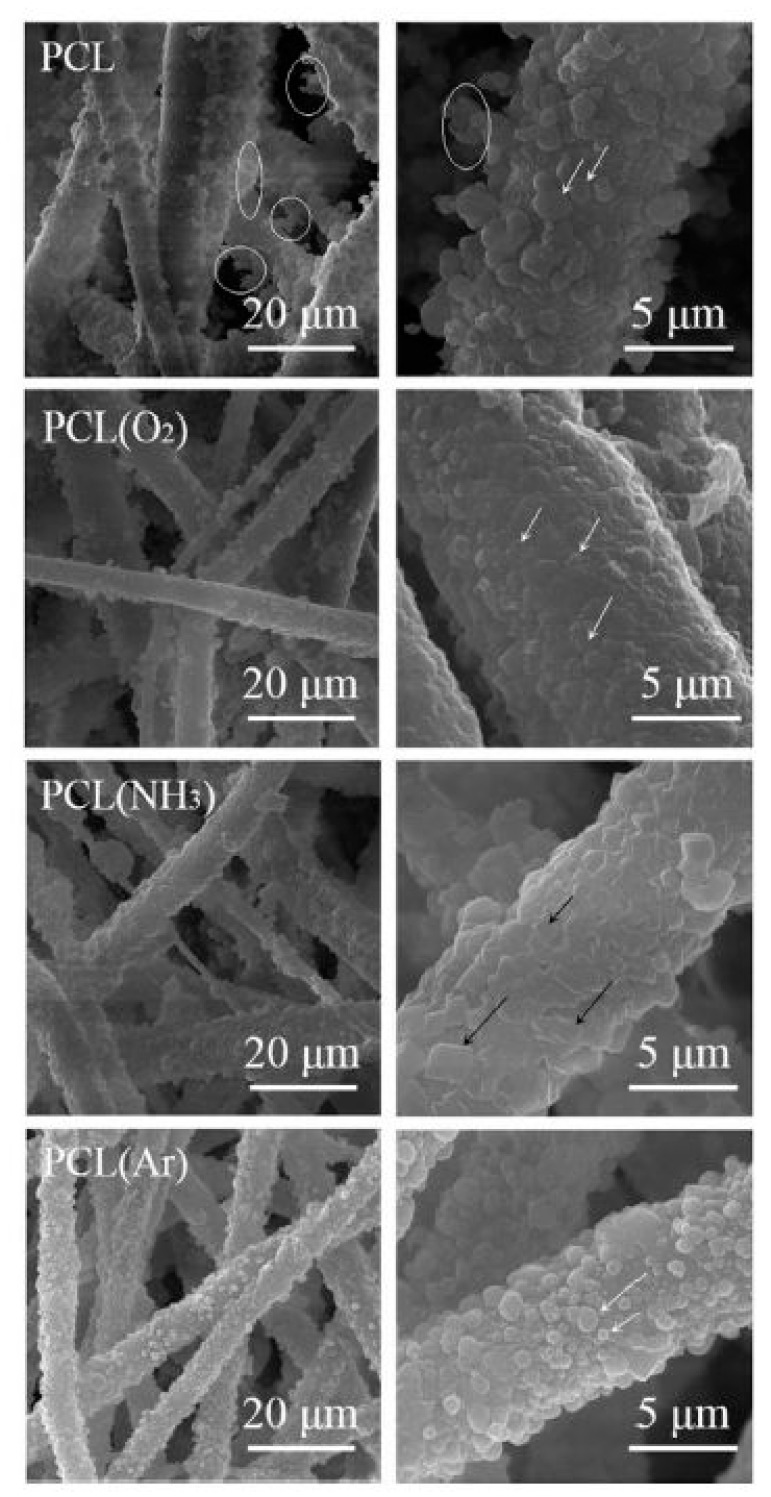
SEM images of CaCO_3_ enrichment on untreated, O_2_, NH_3_, and Ar plasma-treated PCL nanofibrous scaffolds at different magnifications—reproduced with permission from [244]. Copyright Royal Society of Chemistry, 2018.

Ivanova et al. [244] have examined in detail the enrichment of PCL nanofibrous scaffolds with CaCO_3_ on untreated and different plasma-treated samples. After performing plasma activation experiments in O_2_, NH_3_ and Ar atmospheres, the scaffolds were immersed in a CaCl_2_ solution, followed by the addition of a Na_2_CO_3_ solution to the system. This wet precipitation method was found to result in the crystallization of CaCO_3_ particles on the PCL scaffolds. SEM images of the differently prepared CaCO_3_-enriched PCL nanofibrous scaffolds can be found in Figure 23. As shown in this figure, there are clearly visible differences in the granule dispersion and CaCO_3_ coating homogeneity on the different PCL scaffolds under study. For the untreated and Ar plasma-exposed PCL sample, multiple CaCO_3_ agglomerates can be seen on top of the nanofiber surfaces (the areas inside the circles in Figure 23). In contrast, in the case of the deposited coating layer on the O_2_ plasma-treated PCL scaffold, a rather smooth and uniform surface was noticed. For this sample, the CaCO_3_ coated layer consisted of agglomerates of spherical porous CaCO_3_ microparticles attributed to be vaterite (white arrows in Figure 23). In contrast, for the NH_3_ plasma-treated sample, the coating contains cubic CaCO_3_ particles (black arrows in Figure 23), indicative of the calcite phase. These differences in coating morphology and phase composition were explained by the authors by the different surface composition of the plasma-modified PCL scaffolds. The involved researchers presumed that the O_2_ plasma treatment resulted in the production of a large number of active growth sites for the nucleation of vaterite granules, which lead to a more homogeneous and uniform coating morphology than obtained with the Ar and NH_3_ plasma modifications. In contrast, after NH_3_ plasma treatment, the PCL surface becomes saturated with amino groups, which induces the crystallization of CaCO_3_ into calcite. Based on their results, the authors concluded that the CaCO_3_ enriched O_2_ plasma-treated PCL scaffold shows the highest potential for bone TE applications.

Besides CaCO_3_, nanohydroxyapatite (n-HA) has also been coated onto the surface of plasma-activated nanofibrous scaffolds for bone TE purposes. Seyedjafari et al. [296] have focused on this particular topic and have examined the capacity of their fabricated PLLA scaffolds for bone formation in vitro using USSCs under osteogenic induction and in vivo after subcutaneous transplantation in mice. In a first step, the authors performed an O_2_ plasma treatment for 5 min on electrospun PLLA scaffolds, after which the samples were immersed in a 1% (*w/v*) solution of n-HA in deionized water. Based on SEM imaging and an MTT assay, the authors concluded that the plasma-treated and n-HA-coated PLLA scaffolds were observed to support adhesion, elongation and proliferation of USSCs. During osteogenic differentiation, remarkably higher levels of alkaline phosphatase (ALP) activity (a major osteogenic marker), biomineralization and bone-linked gene expression were seen on n-HA/PLLA in comparison with plasma-treated PLLA scaffolds. Interestingly, the coating of n-HA on the surface of electrospun PLLA nanofibers was also found effective for bone formation within 10 weeks after subcutaneous implantation, which was not the case when using plasma-activated PLLA scaffolds. Consequently, the authors suggested that the n-HA directly coated on the surface of nanofibers can induce ectopic bone formation in vivo in the absence of exogenous inductive agents or cells [297].

### 4.5. Plasma Polymerization

Besides plasma activation, which is by far the most commonly applied plasma-based method to nanofibrous TE scaffolds, some authors have also conducted plasma polymerization experiments on electrospun nanofibers [297,298,299,300,301]. In this case, monomer molecules in the vapor phase (typically carried by an inert gas flow) are introduced in the active plasma region. Precursor activation/fragmentation will then occur in the plasma discharge due to interactions between the active plasma species and the precursor molecules. These reactive precursor fragments can, in turn, recombine into higher molecular weight compounds (so-called plasma polymers) either on the sample surface or in the active plasma zone before condensing onto the sample surface. It is also noteworthy to mention that plasma polymerization can occur with multiple monomers in the vapor phase, even if they do not contain unsaturated bonds or cyclic structures [232,299,300]. The mechanism of plasma polymerization has already been well-described in specific reviews on this topic and will therefore not be discussed further here [301,302].

In 2014, Safoji et al. [298] were one of the first to use a plasma polymerization approach on electrospun nanofibrous scaffolds envisioning TE applications. These authors exposed polyethylene terephthalate (PET) nanofibers to a low-pressure RF discharge (type CCP) sustained in a mixture of ethylene (C_2_H_4_) and NH_3_ using a mild plasma power of 10 W for a duration of 15 min. These plasma conditions were found to result in the deposition of an NH_2_-rich plasma-polymerized coating on the surface of the electrospun scaffolds. SEM imaging also revealed that upon plasma polymerization, the mean PET fiber diameter was only increased by 16 nm suggesting the deposition of only a very thin NH_2_-rich coating on the PET nanofibers. Consequently, also the porosity of the nanofibrous PET scaffolds did not significantly change after the plasma coating. The authors also compared the behavior of human umbilical vein endothelial cells (HUVEC) on the untreated and plasma-coated PET scaffolds and observed that the coating significantly improved the adhesion and growth of HUVECs. Additionally, also the resistance of HUVECs to flow-induced shear stress was found to be significantly improved by the plasma-polymerized coating. The properties of the plasma-coated PET scaffold thus support the production of desired confluent HUVEC monolayers on the top surface, contrary to conventional vascular grafts, where cells infiltrate inside the material. These plasma-coated scaffolds are thus highly useful for the pre-endothelialization of the luminal side of small-diameter vascular prostheses.

**Figure 24:**
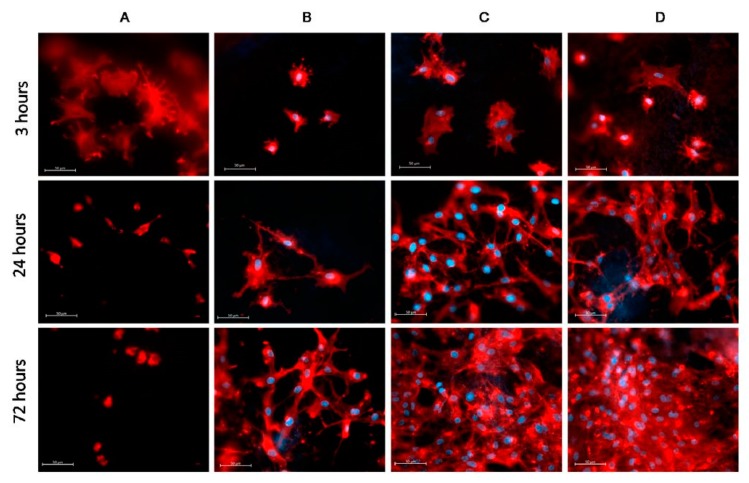
Adhesion of MSCs on the surface of untreated PCL (**A**), COOH-coated PCL (**B**), COOH-coated PCL with physically adsorbed PRP (**C**) and COOH-coated PCL with covalently immobilized PRP (**D**). All images were taken with a magnification of 40× and the scale bar corresponds to 50 µm—reproduced from [294,296]. Copyright Wiley, 2007.

Instead of using a plasma sustained in a C_2_H_4_/NH_3_ mixture, Solovieva et al. [299] applied a low-pressure RF discharge (type CCP) sustained in a mixture of Ar, C_2_H_4,_ and CO_2_ to modify the surface properties of PCL nanofibrous scaffolds. Using this approach, the authors were able to deposit a COOH-rich coating, containing 0.57 at% of COOH groups, on the PCL samples. In the next step, platelet-rich plasma (PRP) was physically adsorbed or covalently immobilized on the COOH-coated PCL scaffolds. The authors examined the biocompatibility of the three types of PCL scaffolds under study by investigating the adhesion, proliferation and viability of human mesenchymal stromal cells (MSCs) on all sample types. Figure 24 shows fluorescent images of MSCs adhering to the differently prepared PCL scaffolds after staining the actin filaments of the cytoskeleton with phalloidin (red) and the cell nuclei with Hoechst 33342 (blue). This figure clearly shows that the MSCs seeded on untreated PCL showed poor coloring of the cytoskeleton after 3 h. The cells were trying to attach to the hydrophobic PCL surface, causing the creation of lamellipodia and actin-rich filopodia. However, the lack of proper adhesion with the untreated PCL scaffold negatively influenced the survival and proliferation of MSCs resulting in poor cell survival on untreated PCL after 72 h. On the COOH-coated sample, the adhesion of MSCs was characterized by smaller spreading areas, but in contrast to the untreated sample, the cells adhered better and showed a considerably distinct network of actin filaments 72 h after cell seeding. Figure 24 also reveals that the best MSC adhesion was observed on the PRP-coated samples: in this case, the MSCs showed an excellent spreading and multiple actin-rich contact points with the surface as well as an obvious network of stress-fibrils.

Cell proliferation and viability studies further revealed that the PRP-coated samples exhibited superior levels of cell proliferation and cell viability. These enhanced cellular responses can be described by the presence of a high amount of PRP fibronectin at the PCL scaffold surface, which is known to strongly enhance cell adhesion, migration, and proliferation [296].

Very recently, Vozzi et al. have also examined the plasma polymerization of acrylic acid (CH_2_ = CHCOOH) on a polyester urethane (PUR) scaffold mimicking cardiac tissue properties in an effort to deposit a COOH-rich coating on nanofibrous samples [300]. A multi-step process was applied by these authors: (1) Ar plasma activation for 1 min, (2) plasma polymerization using a plasma sustained in acrylic acid vapor for 15 min and (3) covalent immobilization of fibronectin on the plasma-coated scaffolds. The deposition of the coating and the protein immobilization were confirmed by FTIR and XPS and the thickness of the deposited COOH-rich coating was found to be only a few nanometers. In the next stage, primary rat neonatal cardiomyocytes were seeded on the fibronectin-functionalized scaffolds and were found to show a high survival rate and a stable beating activity. The signal transduction activity of these scaffolds was also examined and was found to be higher when the cardiomyocytes were cultured on the fibronectin-coated sample compared to TCPS. Real-time polymerase chain reaction analysis also showed a significant modulation at 14 days of cardiac muscle and hypertrophy-specific genes in the fibronectin-functionalized PUR scaffolds, thereby confirming an incipient process of cardiomyocyte maturation [300]. A similar plasma-based strategy as mentioned above was followed by Ko et al., in an effort to deposit COOH-rich coatings on PCL nanofibrous scaffolds [248]. XPS analysis revealed the incorporation of oxygen-containing groups at the acrylic acid plasma-treated PCL surfaces, however, no information is given on the specific number of COOH-groups. Additionally, the mean PCL fiber diameter was also found to decrease upon acrylic acid plasma treatment suggesting that instead of a plasma polymerization process, mainly nanofiber functionalization in combination with etching occurred. The proliferation and differentiation of pre-osteoblast cells (MC3T3) were also examined on the COOH-functionalized samples and compared with the cell behavior on O_2_ plasma-treated PCL scaffolds. MC3T3 adhesion and proliferation (up to 6 days after culturing) were found to be similarly increased for both applied plasma treatments compared to the untreated PCL sample. However, the ALP activity, indicative of osteogenic differentiation, after 8 days of cell culture was found to be higher on the acrylic acid-functionalized PCL scaffolds than on the O_2_ plasma-treated PCL sample.

**Figure 25:**
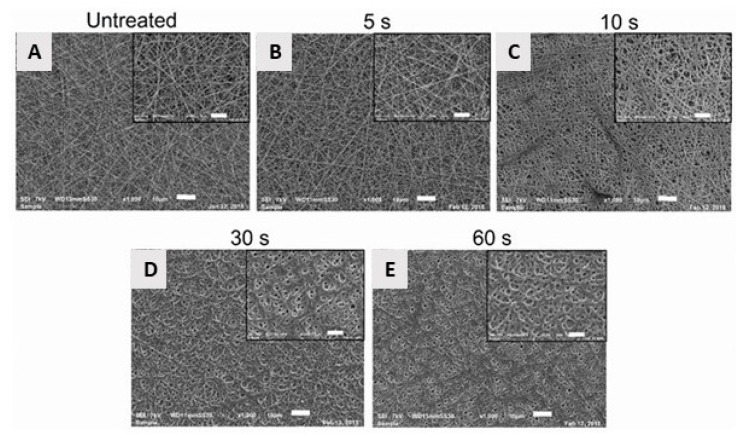
SEM images of PCL nanofibers before (**A**) and after 1-propanethiol plasma polymerization with a plasma exposure time of 5 s (**B**), 10 s (**C**), 30 s (**D**) and 60 s (**E**) (scale bars = 5 and 10 µm)—reproduced with permission from [301]. Copyright Elsevier, 2019.

A final example of plasma polymerization experiments on nanofibrous scaffolds can be found in the recently published paper written by Asadian et al. [301]. These authors have focused on the deposition of a thiol (SH)-rich coating on electrospun PCL scaffolds by performing plasma polymerization experiments using 1-propanethiol (CH_3_CH_2_CH_2_SH) as precursor monomer. A low-pressure RF discharge (type ICP) was used with plasma exposure times varying between 5 and 60 s at a discharge power of 100 W. Figure 25 shows the SEM images of untreated PCL nanofibers (A) and after a plasma deposition time of 5, 10, 30 and 60 s in Figure 25B–E respectively. After a short deposition time of 5 s, the nanofibrous PCL morphology was noted to be quite close to the morphology of the untreated PCL nanofibers. Additionally, the average PCL fiber diameter was slightly larger for this sample than for the pristine PCL scaffold, evidencing the deposition of an approximately 15 nm thick coating on the PCL nanofibers after 5 s. By carrying out a very short plasma polymerization step, it was thus possible to keep the bio-mimicking nanomorphology of the PCL nanofibers. On the contrary, when further increasing the plasma exposure time, considerable changes in the nanofibers’ morphology were seen. At plasma exposure times ≥10 s, the coating was non-homogeneously deposited (higher deposition on specific areas of the nanofibers) as can be found in Figure 25C–E. Moreover, the original nanofibrous morphology of the untreated PCL nanofibers also gradually vanished by enhancing the plasma exposure time as considerably thicker coatings were deposited in this case. Consequently, the authors decided to use a pre-defined plasma polymerization time of 5 s for their remaining tests. Upon plasma polymerization, the surface hydrophilicity was found to be largely unaffected, while XPS analysis revealed the introduction of sulphur-containing groups at the PCL nanofiber surfaces. The number of SH-functional groups was also quantified using XPS chemical derivatization and was equal to 1.8 ± 0.5% when using a plasma exposure time of 5 s. In the final stage of their research, the authors examined the in vitro adhesion and proliferation of bone marrow stem cells (BMSTs) and observed superior MBST adhesion and proliferation on the thiol-coated samples compared to pristine PCL.

As evidenced from the above paragraphs, only a few studies are currently published on the deposition of plasma polymeric coatings on TE scaffolds, however, the results acquired in these studies show great potential for plasma polymerization. As such, it is anticipated that more and more research will emerge in the near future on the use of plasma polymerization approaches for TE applications.

## 5. Conclusions and Outlook

The main goal of this review was to give a comprehensive overview of the manufacturing strategies (including thermally-induced phase separation, molecular self-assembly, and electrospinning) of thermoplastic biodegradable nanofibrous scaffolds within the field of TE and the added value NTP brings compared to other established techniques. The majority of the reviewed work focused on the effects NTPs fueled by inert (Ar, He) and/or reactive (Air, O_2_, N_2_, NH_3_…) discharge gases induced on the nanofiber scaffolds and their subsequent in-vitro performance. A variety of cell-types (fibroblasts, osteoblasts, keratinocytes, stem cells…) have been used to study the positive effects plasma treatments had on cell-surface (adhesion, proliferation, and migration) and cell-protein interactions (immobilization efficiency), for which potential applicability could be found in both hard (bone, cartilage) and soft (skin, nerve, vein…) TE. Despite plenty of positive and promising results, the following concluding remarks should be made:

Almost all nanofibrous supports have a pseudo-2D geometry, a condition that stands far from the actual structures required for TE. Reshaping nanofiber sheets is challenging, as they are easily damaged, and they tend to charge when removed from the collector plate, resulting in spontaneous folding. Some researchers have found ways to deposit them in a tubular fashion by adapting the geometry of the collector plate, making them suitable for vein and nerve tissue regeneration. Still, the highest potential lies in the combination of additive manufacturing technology with electrospinning, a process that has emerged recently and is referred to in literature as direct writing electrospinning, which allows for layer-by-layer deposition of electrospun bundles. In combination with NTP, it is expected that this technology will continue to disrupt the current research lines and will lead to the next generation of nanofibrous substrates for TE.

In most of the collected literature, notably little attention was given to the stability of the plasma treatment during storage, despite the known fact that plasma-activated thermoplasts tend to lose their treatment efficiency over time, a phenomenon known as aging or hydrophobic recovery. For nanofibers, these aging phenomena are expected to be even more pronounced, as the active surface area is orders of magnitude higher compared to non-porous surfaces. This susceptibility to aging could prevent efficient water penetration into the fibrous structure, thereby negatively altering the in-vitro performance.

Although plenty of data is available on possible effects of NTP on cell adhesion and proliferation, very little information can be found on the induced effects on the metabolic activity of the seeded cells; e.g., almost no studies are available that look at the impact of the plasma surface modifications on gene expression. This sort of data is more readily available for other surface modification techniques and is considered vital to understand the exact impact NTP can have on specific cell-surface interactivity.

Another surprising observation is the lack of in-vivo data of plasma modified nanofibrous structures, even though for other types of scaffolds big discrepancies were described between in-vitro and in-vivo performance. Without the necessary in-vivo studies, it seems that most of the aforementioned studies will never surpass the label of “potentially useful” rather than finding their way to the clinical level. Hopefully, this void in knowledge will be filled in the next few years, leading to a selection of plasma-enhanced nanofibrous structures becoming available on the market.

Finally, the review paper also indicated that the use of plasma polymerization has not yet widely spread within the nanofiber community, despite its many advantages over plasma activation: higher functional group density, better stability over time and overall better in-vitro performance. Although one has to be very careful in the selection of the plasma processing parameters to avoid fiber degradation/deformation and pore closure, it is expected that plasma coated nanofibers will inevitably play a more prominent role in the near future.

These remarks clearly show that there still remains a lot of unexplored potentials and it seems that many research groups are only now starting to realize the added value NTP brings to nanofiber-based TE. It is therefore expected that in the future, the research volume dedicated to NTP-enhanced nanofibers will only increase, hopefully leading to products that can have a positive impact on a clinical level.

## Figures and Tables

**Table 1:** Advantages and disadvantages of different techniques for polymer nanofibrous scaffold fabrication.

Technique	Ease of Processing	Advantages	Disadvantages
Phase separation	Easy	3D pore arrangement Pore size and shape are highly controllable Tailorable mechanical properties	Complex procedures Lack of control of fiber arrangement (dimension and orientation) Low yield Limited range of polymers can be used
Self-assembly	Difficult	Easy incorporation of cells during fiber formation 3D pore arrangement Injectable in vivo Great control of 3D shape Easy to fabricate nanofibers on a low scale	Very complex procedure Limited fiber diameter and short fibers Lack of control of fiber orientation and arrangement Low yield Limited range of polymers can be used
Electrospinning	Easy	Simple, cost-effective technique Long continuous fibers Possibility to use a wide range of materials Controllable process parameters to have various fiber diameters in different orientations Well-stablished and characterized technique Tailorable mechanical properties	Poor cell infiltration into the scaffolds’ core Mostly 2D scaffolds Usage of toxic solvents Difficult to control pore size and shape

**Table 2:** Examples of the fabrication of electrospun PLLA scaffolds for TE applications.

Solvent	PLLA Concentration	Applied Voltage	Solution Flow Rate	Spinneret-Collector Distance	Collector Type	Nanofiber Morphology	Average Nanofiber Diameter	TE Application	Ref.
Dichloromethane (DCM)	4 wt%	20–30 kV	14 µL/min	15 cm	Flat plate	Randomly oriented, porous, bead-free PLLA nanofibers	775 ± 294 nm	Bone TE	[157,158]
DCM/*N,N*-dimethylformamide (DMF) (9:1 *v/v*)	5 wt%	15 kV	2 mL/h	15 cm	Flat plate	Randomly oriented, smooth, bead-free PLLA nanofibers	500 ± 110 nm	Bone, blood vessels or cartilage	[159]
Chloroform (CHL)/Methanol (4:1 *v/v*)	5 wt%	20 kV	1 mL/h	12 cm	Drum rotating at 400 rpm	Randomly oriented, smooth, bead-free PLLA nanofibers	563 ± 177 nm	Bladder smooth muscle	[160]
CHL/DMF (4:1 *v/v*)	3 wt%	20 kV	1 mL/h	12 cm	Drum rotating at 400 rpm	Randomly oriented, smooth, bead-free PLLA nanofibers	171 ± 62 nm	Bladder smooth muscle	[160]
DCM/DMF (60:40 *v/v*)	10 *w/v*%	15 kV	0.5 mL/h	10 cm	Flat plate	Randomly oriented, smooth, bead-free PLLA nanofibers	360 ± 174 nm	Not specified	[161]
DCM/DMF (60:40 *v/v*)	10 *w/v*%	15 kV	0.5 mL/h	10 cm	Flat collector with 2 dielectric boards	Aligned, smooth, bead-free PLLA nanofibers	487 ± 167 nm	Not specified	[161]
HFIP	10 wt%	2 kV/cm	100 µL/min	15 cm	Flat plate	Randomly, oriented, smooth, bead-free PLLA nanofibers	1 µm	Heart tissue constructs	[162]

**Table 3:** Some examples of the fabrication of electrospun PDLA scaffolds for TE applications.

Solvent	PDLA Concentration	Applied Voltage	Solution Flow Rate	Spinneret-Collector Distance	Collector Type	Nanofiber Morphology	Average Nanofiber Diameter	TE Application	Ref.
DMF	35 wt%	20 kV	20 µL/min	15 cm	Rotating drum	Randomly oriented, smooth, bead-free PDLA nanofibers	Not mentioned	Not specified	[163]
Acetone/DMF (50:50 *v/v*)	10 *w/v*%	20 kV	1 mL/h	15 cm	Flat plate	Randomly oriented, smooth, defect-free PDLA nanofibers	210 ± 36 nm	Not specified	[164]
Acetone/Dimethylacetamide (DMAc) (50:50 *v/v*)	10 *w/v*%	20 kV	1 mL/h	15 cm	Flat plate	Randomly oriented, smooth, defect-free PDLA nanofibers	357 ± 75 nm	Not specified	[164]
CHL/Acetone (2:1 *v/v*)	5 wt%	18 kV	Not specified	15 cm	Flat plate	Randomly oriented, smooth, bead-free PDLA nanofibers	847 ± 232 nm	Not specified	[165]

**Table 4:** Different electrospun PCL scaffolds for TE applications.

Solvent	PCL Concentration	Applied Voltage	Solution Flow Rate	Spinneret-Collector Distance	Collector Type	Nanofiber Morphology	Average Nanofiber Diameter	TE Application	Ref.
CHL	10 wt%	13 kV	0.1 mL/min	Not specified	Flat plate	Randomly oriented, irregularly shaped PCL nanofibers	400 ± 200 nm	Bone regeneration	[167]
Acetone	7.5 wt%	14–30 kV	1 mL/h	10 cm	Flat plate	Randomly oriented, smooth, bead-free PCL nanofibers	478 nm	Bone regeneration	[168]
CHL/DMF (7.5:2.5 *v/v*)	5 wt%	25 kV	0.5 mL/h	18 cm	Cylindrical drum rotating at 250 rpm	Randomly oriented, smooth, bead-free PCL nanofibers	450–800 nm	Cardiovascular TE	[169]
CHL/DMF (7.5:2.5 *v/v*)	5 wt%	25 kV	0.5 mL/h	18 cm	Cylindrical drum rotating at 2800 rpm	Aligned, smooth, bead-free PCL nanofibers	450–800 nm	Cardiovascular TE	[169]
CHL/DMF (1:1 *v/v*)	15 *w/v*%	1.1 kV/cm	0.375 mL/h	Not specified	Double-bevel collector	Nanofiber centimeter-scaled scaffolds consisting of uniaxially aligned, bead-free PCL nanofibers	220 ± 22 nm	Not specified	[170]
DCM	10 wt%	20 kV	8 mL/h	16.5 cm	Flat plate	Randomly oriented, smooth, bead-free PCL nanofibers	2.4 ± 0.43 µm	Heart valve engineering	[171]
Acetic acid (AA)/deionized water (9:1 *v/v*)	20 wt%	25 kV	1.3 mL/h	8 cm	Flat plate	Randomly oriented, smooth, bead-free PCL nanofibers	278 ± 14 nm	Not specified	[172]
Formic acid (FA)/AA (9:1 *v/v*)	14 *w/v*%	32–33 kV	0.7 mL/h	15 cm	Cylindrical drum rotating at 300 rpm	Randomly oriented, smooth, bead-free PCL nanofibers	107 ± 20 nm	Not specified	[106]
FA/AA (9:1 *v/v*)	14 *w/v*%	32 kV	0.5–0.7 mL/h	20 cm	Cylindrical drum rotating at 3000 rpm	Aligned, smooth, defect-free PCL nanofibers	114 ± 24 nm	Not specified	[119]

**Table 5:** A selection of PLGA nanofibrous TE scaffolds obtained using solvent electrospinning.

Solvent	PLGA Concentration	Lactic Acid/Glycolic Acid Ratio	Applied Voltage	Solution Flow Rate	Spinneret-Collector Distance	Collector Type	Nanofiber Morphology	Average Nanofiber Diameter	TE Application	Ref.
DMF	30 wt%	75:25	2 kV/cm	40 µL/min	15 cm	Flat plate	Randomly oriented, smooth, bead-free PLGA nanofibers	Not specified	Not specified	[148]
CHL	15 wt%	50:50	17 kV	4 mL/h	7 cm	Flat plate	Randomly oriented, smooth, bead-free PLGA nanofibers	760 nm	Not specified	[174]
CHL/DMF (3:1 *v/v*)	20 wt%	85:15	15 kV	0.6 mL/h	16 cm	Flat plate	Smooth PLGA nanofibers randomly oriented with some beads	164 ± 12 nm	Tendon repair	[175]
THF/DMF (1: 1 *v/v*)	5 *w/v*%	85:15	18 kV	Not specified	20 cm	Flat plate	Randomly oriented, smooth, defect-free PLGA nanofibers	Diameters ranging from 500 to 800 nm	Cartilage TE	[21]
HFIP	15 wt%	50:50	17 kV	4 mL/h	7 cm	Flat plate	Randomly oriented, smooth, bead-free PLGA nanofibers	270 nm	Not specified	[174]
HFIP	7 wt%	10:90	12 kV	1 mL/h	10 cm	Rotating Teflon tube with diameter of 1.27 mm	Nanofibrous conduit (length:14 mm) consisting of randomly oriented smooth PGLA nanofibers	Not mentioned	Nerve regeneration	[137]
HFIP	10 wt%	10:90	2 kV/cm	100 µL/min	15 cm	Flat plate	Randomly oriented, smooth, bead-free PLGA nanofibers	Not specified	Not specified	[148]

**Table 6:** Comparison of various plasma set-ups.

Plasma Set-Up	Operating Frequency	Properties
DBD	Hz-kHz	Insulation needed for the electrode Electron avalanche Streamer formation Two plasma modes (glow and filamentary)
CCP	MHz	No insulation required for the electrode Electron avalanche Electrons are trapped between the electrode Ions are accelerated towards self-bias electrode
ICP	MHz	Electrode is isolated from the plasma Plasma is generated via a rapidly changing magnetic field

**Table 7:** Summary of in vitro cell studies performed on plasma-activated nanofibrous scaffolds.

	Cell Type	Plasma Source	Gas	Substrate	In Vitro Results	Ref.
Bone	Human-induced pluripotent stem cells (iPSCs)	MW	O_2_	Polyethersulfone (PES)	Enhanced proliferation and osteogenesis	[261]
	Human primary osteosarcoma cells (Saos-2)	RF	O_2_ and Ar	PCL	Improved cell viability and proliferation	[243]
	Mouse osteoblast cells (MC3T3-E1)	RF	Ar/O_2_, NH_3_/O_2_ and N_2_/H_2_	PCL	Improved cell attachment and proliferation	[208]
	MC3T3	RF	O_2_	PCL	Improved cell adhesion and ALP activity	[246]
	Human mesenchymal stem cells (hMSCs)	Not specified	Ar and N_2_	PCL	Improved cell attachmentAccelerated differentiation towards osteoblasts	[262]
	hMSCs	Not specified	He	PCL/CMC	Enhanced osteoinductivity without external osteogenic differential agent, did not support the proliferation	[263]
	hMSCs	RF	O_2_	PolyActive	Significant upregulation of bone sialoprotein and osteonectin expression	[211]
	hMSCs	Not specified	Air	PLGA	Greatly enhances peptide immobilization which increases the ALP activity, calcium content and expression osteogenic markers of collagen type-I, osteocalcin (OC) and osteopontin (OP)	[264]
	hMSCs	RF	O_2_	PLLA	Improved expression of genes associated with osteoblast linkage	[265]
	hMSCs	Not specified	Air	PLLA	Improved cell proliferation, ALP activity and mineralization	[266]
	hMSCs	Not specified	Air	PLLA/PVA	Increases the ALP activity level, protein content and calcium deposition	[267]
Cartilage	Mouse chondrocyte teratocarcinoma-derived cells (ATDC5)	RF	O_2_ and Ar	PCL	Improved cell viability and proliferation	[243]
	Neonatal human knee articular chondrocytes (nHAC-kn)	MW	Ar	Silk fibroin	Improved cell attachment, proliferation and glycosaminoglycan synthesis	[250]
	Schwann cells (RT4-D6P2T)	RF	Air	PCL	Improved cell proliferation	[258]
	MSCs	Not specified	Air	PCL	Improved cell attachment and proliferation, chondro-differentiation in a non-differential medium	[268]
	Mouse lung fibroblasts (L929)	RF	O_2_ and Ar	PCL	Improved cell viability and proliferation	[243]
	Human foreskin fibroblasts (HFFs)	DBD	Ar, N_2_ and He/NH_3_	PCL	Improved cell adhesion and proliferation	[180]
	HFFs	DBD	Ar, N_2_ and He/NH_3_	Chitosan/PEO	Improved cell adhesion and proliferation	[254]
	Normal human epidermal keratinocytes and fibroblasts (NHEKs and NHEFs)	RF	O_2_	Silk fibroin	Improved cell attachment	[249]
Epithelial	3T3 fibroblasts	DBD	O_2_ and NH_3_	PLGA	Improved cell adhesion and proliferation (NH_3_ > O_2_)Improved cell adhesion and spreadingDecreased stress gene expression	[258]
			NH_3_			[259]
	Mouse embryonic fibroblasts (MEFs)	Corona	N_2_	PLLA	More elongated and dendritic cell morphologyImproved cell vitality	[256]
	Bovine aorta endothelial cells (BAECs)	RF	Ar and Ar-NH_3_/H_2_		Improved adhesion, spreading and infiltration	[247]
Stem cells	Porcine mesenchymal stem cells (pMSCs)	RF	O_2_	PLLA	Improved cell adhesion	[250]
	Adipose-derived stem cells (ADSCs)	DBD	Ar and Air	PCL	Improved cell adhesion, proliferation, spreading and viability	[269]
Muscle	Primary porcine smooth muscle cells (SMCs)	RF	Air	PCL	Improved spread-out cell morphology	[250]
	Bovine smooth muscle cells (BSMCs)	RF	Ar and Ar-NH_3_/H_2_		Improved adhesion, spreading and infiltration	[247]
Immune System	Human monocyte	RF	Air	PLLA	Disruption of macrophage polarization balance towards an anti-inflammatory profileImproved cell morphology with filopodia-like and podosome-like structures on plasma-treated samples	[270]
